# Increased Learning and Brain Long-Term Potentiation in Aged Mice Lacking DNA Polymerase μ

**DOI:** 10.1371/journal.pone.0053243

**Published:** 2013-01-03

**Authors:** Daniel Lucas, José M. Delgado-García, Beatriz Escudero, Carmen Albo, Ana Aza, Rebeca Acín-Pérez, Yaima Torres, Paz Moreno, José Antonio Enríquez, Enrique Samper, Luis Blanco, Alfonso Fairén, Antonio Bernad, Agnès Gruart

**Affiliations:** 1 Department of Immunology and Oncology, Centro Nacional de Biotecnología/CSIC, Madrid, Spain; 2 División de Neurociencias, Universidad Pablo de Olavide, Sevilla, Spain; 3 Department of Regenerative Cardiology, Centro Nacional de Investigaciones Cardiovasculares, Madrid, Spain; 4 Development and Cardiac Repair Department, Centro Nacional de Investigaciones Cardiovasculares, Madrid, Spain; 5 Centro de Biología Molecular Severo Ochoa, Campus de Cantoblanco, Madrid, Spain; 6 Instituto de Neurociencias, Consejo Superior de Investigaciones Científicas - Universidad Miguel Hernández, San Juan de Alicante, Spain; 7 Translational Research Platform, Centro Nacional de Investigaciones Cardiovasculares, Madrid, Spain; University of Medicine and Dentistry of New Jersey, United States of America

## Abstract

A definitive consequence of the aging process is the progressive deterioration of higher cognitive functions. Defects in DNA repair mechanisms mostly result in accelerated aging and reduced brain function. DNA polymerase µ is a novel accessory partner for the non-homologous end-joining DNA repair pathway for double-strand breaks, and its deficiency causes reduced DNA repair. Using associative learning and long-term potentiation experiments, we demonstrate that Polµ^−/−^ mice, however, maintain the ability to learn at ages when wild-type mice do not. Expression and biochemical analyses suggest that brain aging is delayed in Polµ^−/−^ mice, being associated with a reduced error-prone DNA oxidative repair activity and a more efficient mitochondrial function. This is the first example in which the genetic ablation of a DNA-repair function results in a substantially better maintenance of learning abilities, together with fewer signs of brain aging, in old mice.

## Introduction

Non-homologous end-joining (NHEJ) is a fundamental pathway for the repair of double-strand breaks (DSB) in mammals. Deficiency in any of the NHEJ core factors results in immunodeficiency, general sensitivity to double-strand-break-inducing agents, and premature cellular senescence [1], [2]. When, due to their structure, broken DNA ends cannot be directly ligated, NHEJ reactions require a variety of accessory factors to pre-process the ends. Three DNA polymerase activities (TdT, Polλ and Polµ), belonging to the PolX family, are involved in this step of the NHEJ reaction in mammals [3]–[6]. The three polymerases participate in the repair of similar DNA intermediates, but due to its unique structural features, Polμ is the only one capacitated to repair DNA breaks whose 3′protrusions have null complementarity [6]–[8].

Recently, the structure of all members of the PolX family has been resolved [9], and a working model for Polµ action during end-bridging of broken DNA ends (Text S1and Figure S1) proposed [9], [10]. According to this model, and considering the ability of Polµ to carry out untemplated deoxynucleotide and ribonucleotide insertions [11], [12], Polµ would behave as an error-prone DNA repair polymerase. It is therefore currently thought that, during the NHEJ reaction, Polµ will be used as a backup to other PolX members that are less prone to introducing mutations in the DNA. Conversely, the analysis of knockout mouse models indicates that only Polµ seems to promote selective accuracy during immunoglobulin kappa recombinatiom [11], as immunoglobulin heavy chain junctions from Polµ-deficient (Polµ^−/−^) animals have shorter length with normal N-additions [13], [14]. Hence, it was proposed that Polµ, Polλ and TdT have non-overlapping functions during immunoglobulin V(D)J recombination [14].

Polµ^−/−^ animals present impaired DSB repair [15], and this in turn causes a significant alteration in hematopoietic homeostasis. During the regular phenotyping of Polµ^−/−^ mice, we realized that they showed remarkably low exploratory behavior after cage change, at different age stages (*P*≤0.05; Text S1 and Figure S2A, B). Moreover, analysis of Polµ^−/−^ mice in the rota-rod test showed significantly better sensorimotor coordination at the three ages analyzed (3, 8, and 18 months old; *P*≤0.05; Text S1 and Figure S2C, D); furthermore, 18-month-old Polµ^−/−^ mice demonstrated that they were able to improve sensorimotor coordination over the 4 days of the test, contrary to age-matched wild-type controls that were unable to do so (Text S1 and Figure S2D). These data suggested improved brain function at old age in Polµ^−/−^ mice. Although Polµ is expressed in the central nervous system [11], its specific function in it remains to be determined. We have analyzed here the consequences of genetic elimination of Polµ in a fundamental aspect of the central nervous system: deterioration of learning capacities in association with aging [16]. Furthermore, we have evaluated young (3-month-old) and aged (18-month-old) wild-type or Polµ^−/−^ mice in associative learning and long-term potentiation (LTP) tests [17].

## Results

### Classical Conditioning of Eyelid Responses in Behaving Wild-type and Polµ^−/−^ Mice

**Figure 1:**
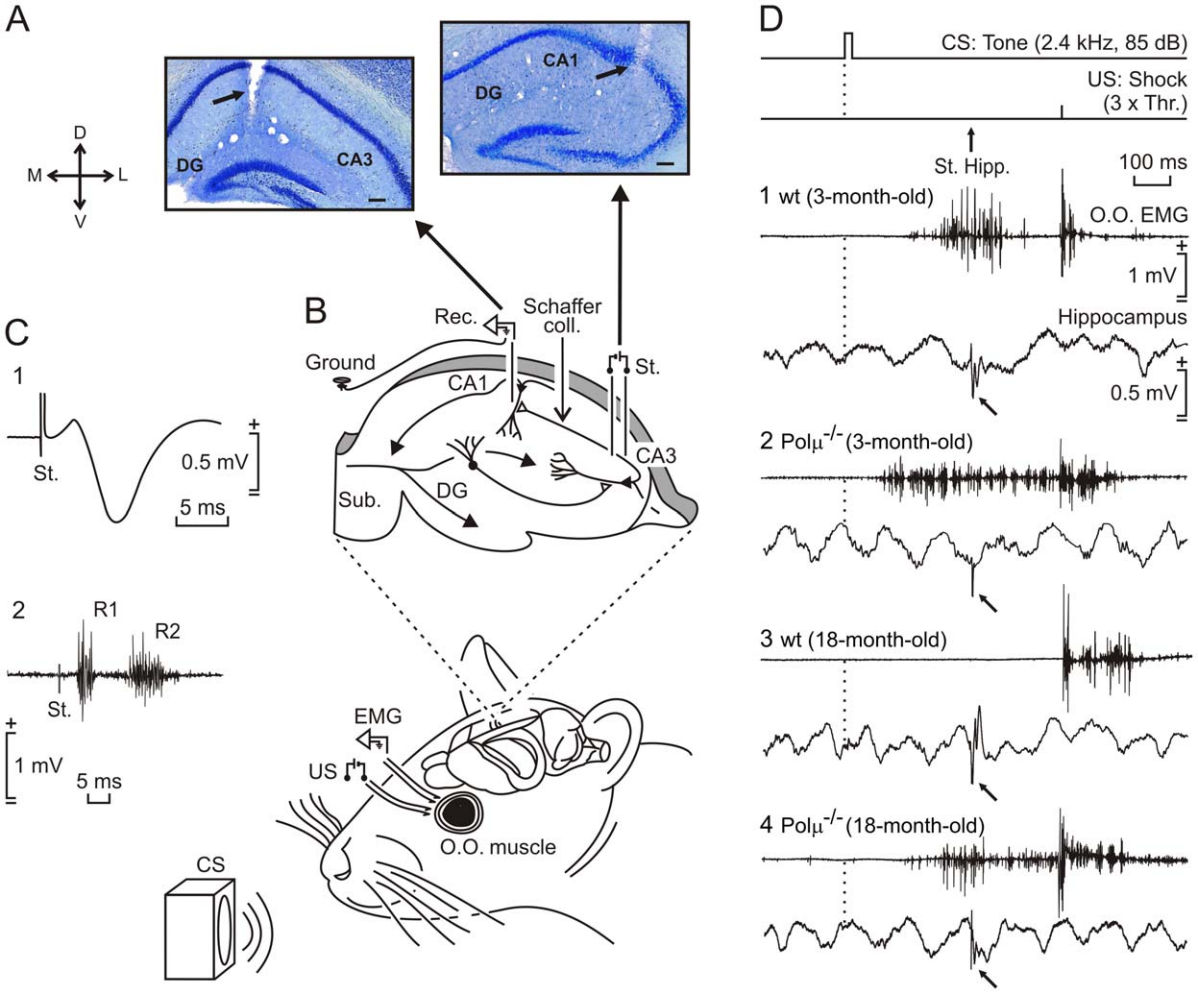
Experimental design for recording activity-dependent synaptic changes at the CA3-CA1 synapse during classical eyeblink conditioning. (**A**) Location of recording (left) and stimulating (right) electrodes implanted chronically in the CA1 and CA3 areas respectively. (**B**) Animals were implanted with electromyographic (EMG) recording electrodes in the orbicularis oculi (O.O.) muscle, and with stimulating electrodes on the supraorbital nerve for presentation of unconditioned (US) stimuli. Conditioned stimulus (CS) consisted of a tone preceding the US by 500 ms. Animals were also implanted with recording (Rec.) electrodes in the CA1 area and with stimulating (St.) electrodes at the ipsilateral Schaffer collaterals. (**C**) Example of fEPSP evoked at the CA3-CA1 synapse in an 18-month-old Polµ^−/−^ mouse (1) and eyeblink evoked in the O.O. muscle by the electrical stimulation of the supraorbital nerve (2). (**D**) The top two traces illustrate the trace conditioning paradigm, and the moment at which a single pulse was presented to Schaffer collaterals (arrow, St. Hipp.). Samples of EMG activity of the O.O. muscle and hippocampal extracellular activity collected from the 9th conditioning session from an animal of each experimental group (1–4). Calibrations in (1) are for all of the records. Note fEPSPs evoked by the pulse presented to Schaffer collaterals (bend arrows).

**Figure 2:**
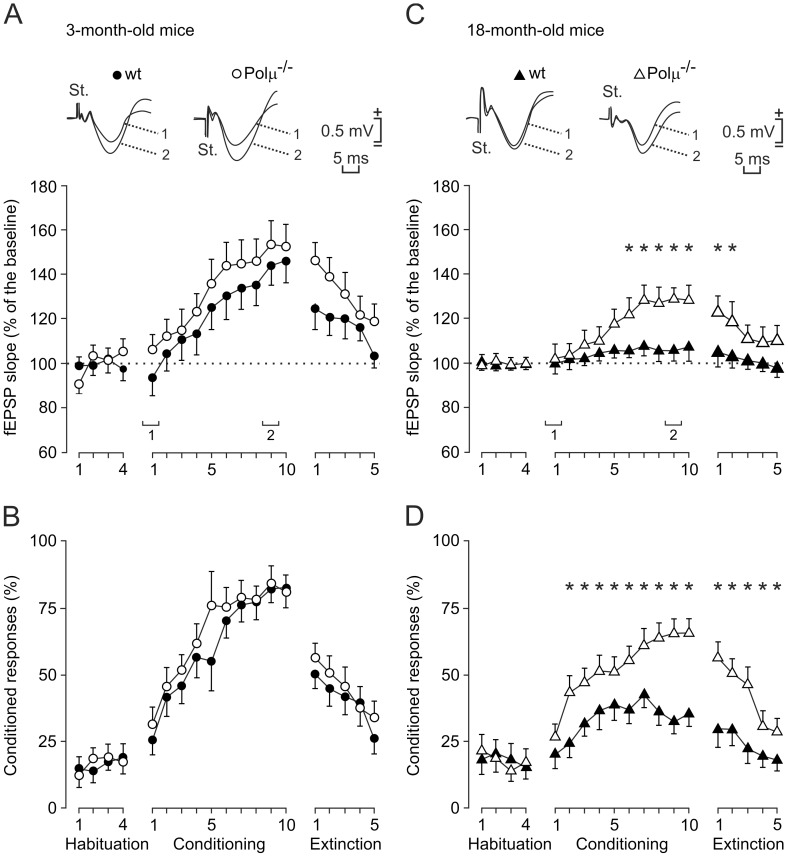
Learning curves and evolution of the evoked field potential (fEPSP) at the CA3-CA1 synapse for young and aged Polµ^−/−^ and wild-type mice, across training. (A, B) Evolution of the fEPSP slope (A) and of the percentage (%) of conditioned responses (CRs, B) for 3-month-old wild-type (black circles) and Polµ^−/−^ (white circles) mice, during habituation, conditioning, and extinction sessions. At the top are illustrated averaged (n = 5) fEPSPs collected from the 1st and 9th conditioning sessions. (C, D) A similar set of data collected from 18-month-old wild-type (black triangles) and Polµ^−/−^ (white triangles) animals. Mean % values are followed by ± SEM. Differences in the percentage of CRs between 18-month-old wild-type and Polµ^−/−^ animals were statistically significant as indicated (*P<0.001, two-way ANOVA). Differences in fEPSP slopes are also indicated, expressed as the % change with respect to mean values collected during the last two-habituation sessions (*P<0.001, two-way ANOVA).

Trace conditioning is a hippocampus-related paradigm of associative learning [18], [19]. Mice are capable of acquiring classically conditioned eyelid responses using trace paradigms [17], [20], [21]. Polµ^−/−^ and wild-type animals (n = 10 per group) were classically conditioned, using a tone as a conditioned stimulus (CS) and an electrical shock presented to the supraorbital nerve as an unconditioned stimulus (US; Figure 1). The percentage of conditioned responses (CRs) for 3-month-old wild-type and Polµ^−/−^ animals was similar (Figure 2B), with a profile equivalent to previous descriptions in mice, using comparable trace conditioning procedures [17], [19]–[21]. Although the learning curve for the Polµ^−/−^ group was steeper than that for wild-type animals (76.5% vs. 55% of CRs by the 5th session, respectively), the two groups reached similar asymptotic values by the last four conditioning sessions (Figure 2B). Both 18-month-old wild-type and Polµ^−/−^ mice reached significantly lower CR values than their corresponding 3-month-old controls for the 5th-10th and for the 2nd-10th conditioning sessions, respectively (F_(18,162)_ = 26.11, *P*≤0.05; Figure 2B, D). However, from the 2nd to the 10th conditioning sessions, 18-month-old Polµ^−/−^ mice presented a learning curve significantly steeper than that of their controls (F_(18,162)_ = 26.11, *P*<0.001; Figure 2D). The values reached during the five extinction sessions were also significantly different (*P*<0.001) among 18-month-old wild-type and Polµ^−/−^ mice (Figure 2D). These differences in the acquisition of CRs cannot be ascribed to changes in age-dependent modifications in eyeblink reflex circuits (Text S1 and Figure S3).

Eyeblink conditioning evokes a change in strength at the hippocampal CA3-CA1 synapse in behaving mice [17]. Hence we studied activity-dependent synaptic changes during associative learning in this model, by the presentation of a single electrical pulse to the Schaffer collateral/commissural pathway 300 ms after CS onset (Figure 1A, B). The electrical stimulation of Schaffer collaterals during the CS-US interval evoked a field excitatory postsynaptic potential (fEPSP) in the CA1 area, as illustrated by representative examples of the four experimental groups (Figure 1D). The slope of the evoked fEPSPs increased progressively during conditioning to about 140% of the baseline during the 5th-10th sessions in 3-month-old Polµ^−/−^ mice and during the 8th-10th sessions in the 3-month-old wild-type mice (Figure 2A). In contrast, 18-month-old Polµ^−/−^ mice displayed significantly steeper slopes than those presented by 18-month-old wild-type mice from the 6th to 10th conditioning sessions, and during the first two extinction sessions (F_(18,162)_ = 19.521, *P*≤0.001; Figure 2C). In all experimental groups, with the exception of 18-month-old wild-type mice, fEPSP changes during conditioning were linearly related to learning evolution (Text S1 and Figure S4). These results demonstrate associative learning in 18-month-old Polµ^−/−^ mice and no learning in 18-month-old wild-type mice.

### Long-term Potentiation of the Hippocampal CA3-CA1 Synapse in Wild-type and Polµ^−/−^ Mice

**Figure 3:**
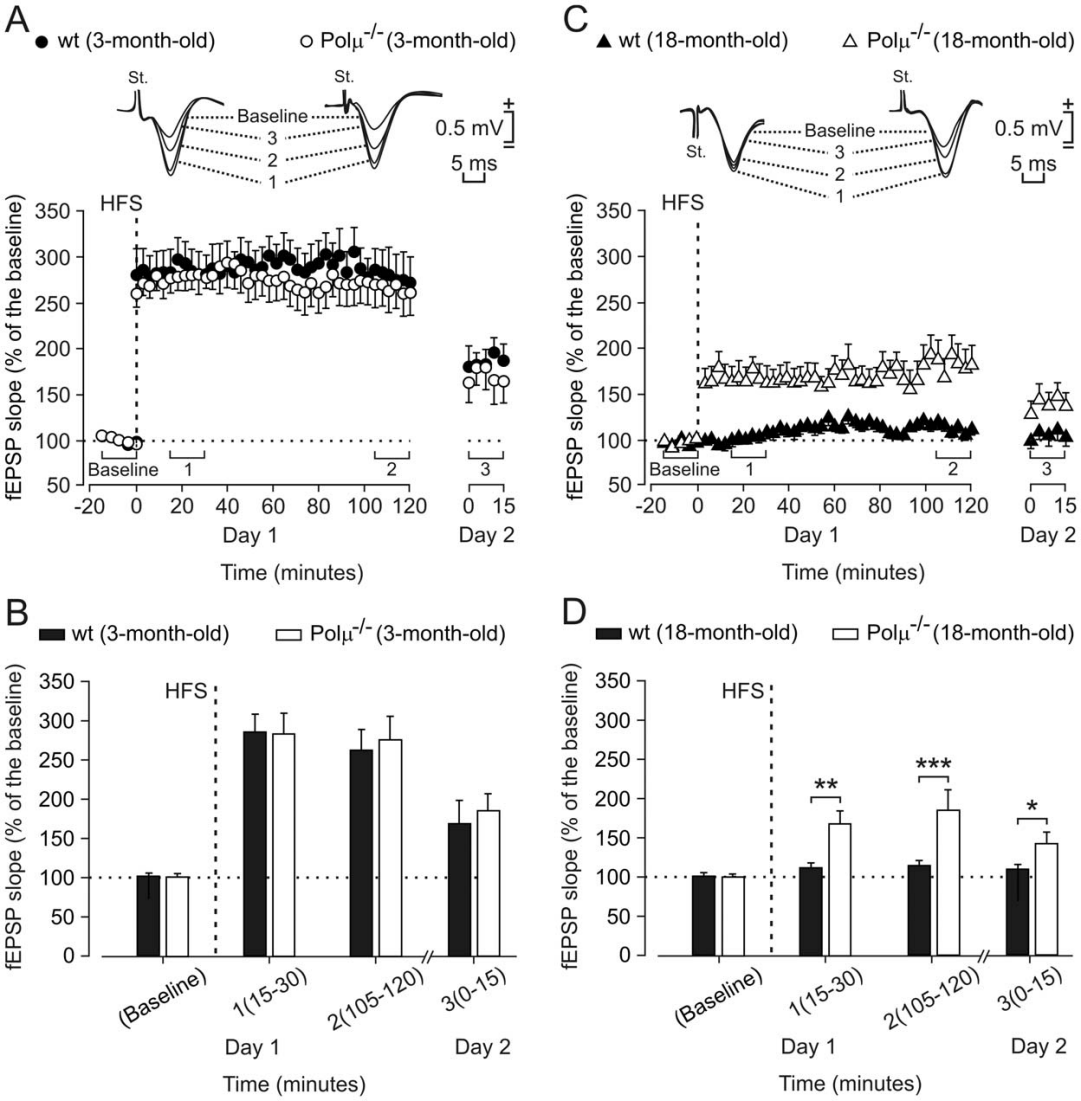
Long-term potentiation (LTP) evoked at the CA3-CA1 synapse in 3- and 18-month-old Polµ^−/−^ and wild-type mice. (**A**) LTP induction in representative 3-month-old control (black circles) and Polµ^−/−^ (white circles) mice. LTP was evoked in CA1 pyramidal cells by HFS of ipsilateral Schaffer collaterals (five 200 Hz, 100 ms trains of pulses at a rate of 1/s. This protocol was presented 6 times in total, at intervals of 1 min). The fEPSP is given as a percentage of the baseline (100%) slope. Records in the inset were collected at the indicated (1–3) times. (**B**) Mean ± SEM fEPSP values 1–15 min before (baseline), and 15–30 min (1), 105–120 min (2), and 24 h (3) after HFS for 3-month-old control (black bars) and Polµ^−/−^ (white bars) animals. No significant differences were observed between LTP evoked in these two groups of animals (*P* = 1, two-way ANOVA). (**C, D**) A similar set of data collected from 18-month-old wild-type (black triangles and bars) and Polµ^−/−^ (white triangles and bars) mice. Note that, in contrast with Polµ^−/−^ mice, 18-month-old controls presented a non-significant increase in fEPSP slopes after the presentation of the HFS protocol (*P<0.05, **P<0.01, ***P<0.001, two-way ANOVA).

LTP is a well-known form of synaptic plasticity that shares many properties with the synaptic potentiation evoked by motor or cognitive learning [17], [21]–[25]. We investigated whether the improved synaptic plasticity observed in the 18-month-old Polµ^−/−^ mice at the hippocampal CA3-CA1 synapse during associative learning could also be detected during LTP evoked by high-frequency-stimulation (HFS) trains applied to Schaffer collaterals; LTP evoked at the CA3-CA1 synapse can last from hours to days [26]. For these experiments, we used an established LTP-evoking protocol (see Methods). Polµ^−/−^ or wild-type (3-month-old) mice showed significant LTP for 24 h (*F*_(36,324)_ = 115.3, *P*<0.001) that was indistinguishable between the two groups. There were no significant differences between the LTP evoked in young (3-month-old) wild-type and Polµ^−/−^ mice (*F*_(49,441)_ = 0.217, *P* = 1; Figure 3A, B). However, and similarly to the results obtained for associative learning (Figure 2), no LTP was evoked in 18-month-old wild-type mice, whereas a clear LTP was evoked in 18-month-old Polµ^−/−^ mice (*F*_(49,441)_ = 4.815, *P*<0.001; Figure 3C, D). Aging has an evident effect on LTP induction, which was significantly smaller in both groups of 18-month-old mice than in their respective 3-month-old matched genotypes (*F*_(49,441)_ = 6.602, *P*<0.001 for wild-type and *F*_(49,441)_ = 7.950, *P*<0.001 for Polµ^−/−^ mice). In marked contrast, the four groups behaved equally in the paired-pulse protocol, a test aimed at detecting the presence of short-term synaptic plasticity (Text S1 and Figure S5). Taken together, these results show that Polµ deficiency enhances the activity-dependent synaptic potentiation, taking place at the CA3-CA1 hippocampal synapse during the acquisition of associative learning in conscious mice [17]. LTP evoked in alert behaving mice by HFS of Schaffer collaterals further confirmed that Polµ plays a definite but negative role in maintaining long-term plastic changes at the CA3-CA1 synapse during the aging process.

### Polµ^−/−^ Mice Brain Shows a Molecular Profile Compatible with a Delayed Aging Phenotype

**Figure 4:**
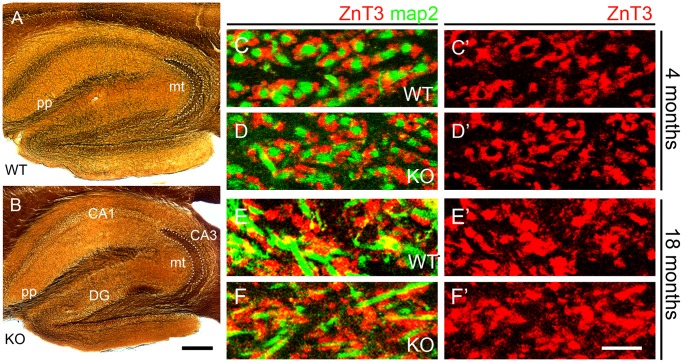
Absence of visible changes in hippocampal circuitry in Polµ^−/−^ mice. (**A, B**) Silver staining shows perforant path axons (pp) and mossy fibers forming axon terminals (mt) in the *stratum lucidum* of CA3 (outlined), in 4-months-old mice. The images do not suggest anatomical differences between wild-type (**A**) and Polµ^−/−^ (KO) mice (**B**). (**C – F’**) Mossy fiber terminals in CA3 *stratum lucidum* immunostained for ZnT3 in wild-type (**C, E**) and Polµ^−/−^ (**D, F**) mice, at 4 months (**C, D**) and 18 months (**E, F**); map2 immunostaining reveals dendrites of CA3 pyramidal cells. The distribution of the ZnT3^+^ mossy fiber expansions was altered with aging, but no differences occurred between wild-type and Polµ^−/−^ (KO) mice of each age. CA1, CA3, areas of the hippocampus proper; DG, dentate gyrus; mt, mossy fiber expansions; pp, perforant pathway. Scale bar, A, B: 250 µm; C–F’: 10 µm.

As Polµ^−/−^ animals show reduced DNA DSB repair capacity and increased sensitivity to ionizing radiation [15], the results described above contrast with those reported for many other DNA-repair defects [27]. To understand the cellular basis of the enhanced neurological functions preservation caused by the lack of Polµ, we have explored the hypothesis that the improved hippocampal function of Polµ^−/−^ mice might correlate with an improved maintenance of neuronal circuits during aging. Classical silver stains (Figure 4A, B) reveal different sets of axonal systems in the trisynaptic circuit of the hippocampus, including the perforant pathway that conveys afferent axons from the entorhinal cortex to the hippocampus proper and the mossy fibers formed by dentate gyrus granule cell axons projecting onto CA3 pyramidal cells. First, we addressed whether young adult, 4-months-old Polμ^−/−^ mice have a normal hippocampal circuitry. No major differences in the distribution of axons of the perforant pathway, or in the area of distribution of mossy fiber terminals in CA3 *stratum lucidum* were found between wild type and Polμ^−/−^ mice at 4 months of postnatal age (Figure 4A, B). The vesicular Zn^2+^ transporter ZnT3 is strongly expressed in synaptic vesicles of the mossy fiber expansions in area CA3 stratum lucidum. We analyzed Znt3^+^ mossy fiber expansions surrounding map2^+^ postsynaptic dendrites of CA3 pyramidal cells in 4- and 18-months-old animals, finding that they were less organized in 18-months-old mice as compared to 4-month-old ones (Figure 4C–F’), a feature possibly associated with aging. However, we observed no apparent changes in these axon terminals induced by the absence of Polµ at both ages (Figure 4C–F’). This suggests that the enhanced functional state of the hippocampal circuits in aged Polµ^−/−^ mice might not be caused by enriched axonal input to at least two elements of the trisynaptic circuit, i.e. the perforant path to dentate gyrus and the mossy fibers to CA3.

No significant differences could be observed in the level of double-strand breaks (DSB; staining for 53BP1 (Figure S6A) or autophagy, evaluated by the expression level of a panel of critical genes (Figure S6B). In addition, Polµ^−/−^ animals seemed not to present better telomere maintenance (Figure S6C, D). Furthermore, we ruled out that the observed effects could be associated with compensatory mechanisms involving the closest partners in DSB repair, namely Polλ and Polβ (Figure S6E).

The adult brain is essentially a post-mitotic organ, and repair of deleterious oxidative DNA damage associated with 8-oxo-guanine (8oxoG) generation is a substantial need. Base excision repair is the main mechanism eliminating 8oxoG from DNA, but several other players have been proposed for the tolerance/repair alternative [28], [29]. 8oxoG behaves as a pre-mutagenic lesion; when used as template by a repair polymerase activity, 8oxoG dictates not only the insertion of a correct dCTP residue (when 8oxoG is in *anti*-orientation), but also has the potential to direct misinsertion of dATP (when 8oxoG adopts a *syn*-orientation), thereby forming an 8oxoG:dAMP mismatch. Contrary to other repair polymerases, such as Polβ or Polλ, purified human Polµ is strongly mutagenic when copying 8oxoG, as dATP or rATP is preferably inserted in front of 8xoG compared with dCTP or rCTP (Figure S7D, E).

**Figure 5:**
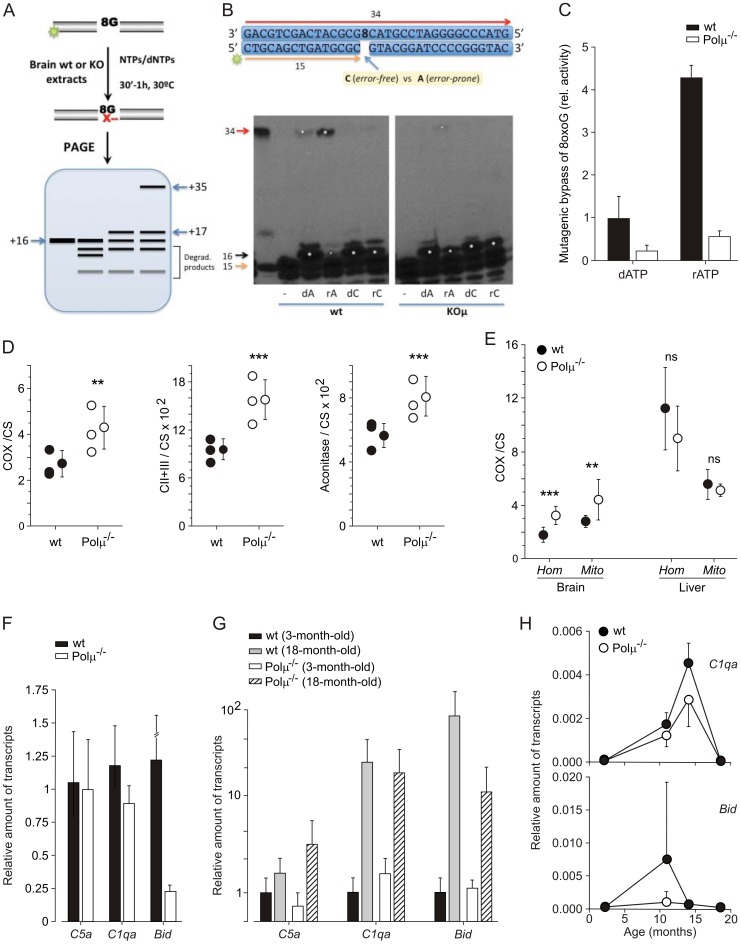
Polµ^−/−^ mice brain shows a molecular profile compatible with a delayed aging phenotype. (**A**, **B, C**) Evaluation of 8oxoG bypass repair activity (tolerance) in brain (wild-type vs. Polµ^−/−^) extracts, using the indicated labeled-template primer (**B**). After incubation (30′–1 h, 30°C) with the clarified extracts (10 µg), with the addition of the indicated nucleotides (50 µM) and 1 mM MnCl_2_, products were recovered and resolved in 20% PAGE. The scheme (**A**) illustrates the mobility of the different expected products: the original labeled primer (+15) could appear intact or degraded by endogenous nucleases (degraded products); extended primers appear at positions (+16 or +17) if no ligation with the 5′-flank of the gap has occurred (short-patch repair), and at full-length repaired (or mutated) product (+34) if ligation did occur. (**C**). Densitometric analysis of full-length repaired (+34) product was carried out, for the reaction using dATP or rATP, and represented relative to the activity yield by wild-type brain extracts using dATP. (**D, E**) Evaluation of mitochondrial function in whole extracts (Hom) or mitochondrial purified fractions (Mits), in brain and liver of wild-type and Polµ^−/−^ mice. Several relevant parameters (COX/CS, CII+CIII/CS, and aconitase/CS ratios) were monitored in brain (**D**) and compared with activity in liver (**E**). Differences were considered statistically significant at *P<*0.05. *P<0.01; **, *P*<0.001; ***, *P*<0.0001 and ns, non-significant. (**F, G, H**) qRT-PCR analysis (**F**) of genes found to be differentially expressed in brain of old Polµ^−/−^ mice (Figure S8). Evolution of gene expression at different ages (**G, H**). All qRT-PCR analyses were carried out in triplicate and normalized to (36B4 or β-actin), and represented as the relative level of expression of the indicated gene.

Evaluation of wild-type versus Polµ^−/−^ brain extracts from old (21–23 months) mice demonstrated a similar overall gap-filling activity in both samples (Figure S7A), in agreement with a greater contribution of other repair polymerases from family X, such as Polβ and Polλ, but – strikingly – Polµ^−/−^ extracts presented a significant reduction in mutagenic 8oxoG bypass activity (Figure 5A–C). When the 1nt-gapped DNA substrate contained an 8oxoG residue as template (see a scheme of the reaction in Figure 5A), the two extracts produced a similar level of the immediate insertion step of the short-patch reactions (+16; indicated by a white asterisk in Figure 5B), using either dA or dC; however, a significant difference was demonstrated for the generation of the full-length (+34) “repaired/ligated” product (also indicated by a white asterisk in Figure 5B). That full-length bypass product (in which 8oxoG was tolerated as template) was especially prominent when dATP (error-prone reaction) was provided in comparison with dCTP (error-free reaction), and was mostly Polμ-dependent (Figures 5B, C and S7). By providing either dGTP or dTTP (Figure S7B), Polμ-dependent mutagenic bypass of 8oxoG was also observed, but at a lower level than that obtained with dATP, mimicking the substrate preference observed with purified human Polμ (Figure S7D).

Polμ has an unusually low discrimination between dNTPs and rNTPs, leading to the proposal that the use of ribonucleotide substrates could be advantageous for DNA repair, especially in non-dividing cells where the dNTPs pool could be greatly reduced [12], [30]. Interestingly, when 8oxoG tolerance was evaluated in wild-type versus Polµ^−/−^ brain extracts using ribonucleotides (rATP versus rCTP), the 34-mer full-length bypass product was preferentially obtained with rATP (mutagenic), and the reaction was about 4-fold more efficient than that with dATP (Figure 5B, C). As shown with deoxynucleotides, the mutagenic (rATP) bypass product was considerably reduced in Polµ^−/−^ brain samples (about 7-fold lower). By providing either rGTP or UTP, Polμ-dependent mutagenic bypass of 8oxoG was also observed (Figure S7C), but at a lower level than that obtained with rATP, again mimicking the substrate preference observed with purified human Polμ (Figure S7E).

These combined data imply that brains from old wild-type animals have a significant mutagenic bypass activity in templates harboring 8oxoG lesions, as the most representative oxidative damage, and that this mutagenic potential is significantly reduced in the absence of Polμ. This revealed global reduction in mutagenic bypass of modeled 8oxoG lesions and can be considered a relevant difference that could have a direct or indirect impact in brain physiology.

Due to the remarkable role of mitochondrial function in oxidative stress and physiological decline [31]–[33] we examined the activity status in old (23–28 months) Polµ^−/−^ or wild-type brain. The analyses did not find differences either in mitochondrial content or in the mtDNA copy-number (Figure S8A, B). However, the results clearly demonstrated that Polµ^−/−^ samples showed a better performance after evaluation of several relevant parameters (COX/CS, CII+CIII/CS, and aconitase/CS ratios) in comparison with equivalent fractions obtained from wild-type brain (Figures 5D and S8C–E); this effect is not global because evaluation of equivalent liver fractions did not reveal such difference (Figure 5E). In conclusion, these results indicating a reduced mutagenic bypass activity on 8oxoG lesions and a more efficient mitochondrial activity in brain of 18-month-old Polµ^−/−^ mice, strongly suggest a plausible impact in the improved brain physiology maintenance in Polµ^−/−^ mice.

Therefore, we analyzed expression levels of a panel of candidate genes, whose increment have been associated with physiological murine and human aging [34] (Figure S9A). In this analysis, only 6 out of 19 genes analyzed showed reduced expression levels in Polµ^−/−^ brains (*Egln3*, *Bid*, *Acin1*, *C1qa*, *Mrps12,* and *Igf1*). The most striking down-regulation in Polµ^−/−^ mice was that of *Egln3/Phd3*, an oxygen sensor. However, because *Egln3* expression could not be detected in the brain or liver (in either wild-type or Polµ^−/−^) before the age of 16 months (and then only in some wild-type mice), we focused on two other candidates: *C1qa* (a member of the complement system) and *Bid* (BH3 interacting domain death agonist), a modulator of pro-apoptotic functions. Both genes showed a clear down-regulation in 18-month-old Polµ^−/−^ brains (Figure 5F), whereas another member of the complement system (*C5a;* used as an internal control) was not significantly modulated. Evolution of the expression levels of these genes during aging in Polµ^−/−^ and wild-type brains (Figure 5G, H) suggest a gene expression pattern partially compatible with a putative retardation in brain aging, although other previously proposed potential aging markers in CNS were not found to be significantly modified or even up-regulated in Polµ^−/−^ old brains, such as P16 (Text S1 and Figure S9A).

Finally, to evaluate the putative involvement of a differential oxidative stress response in the described phenotype, Polµ^−/−^ and wild-type mice were treated with an acute dose of paraquat and brains analyzed. It was demonstrated that 9,365 genes (38.7%) were deregulated as a consequence of the treatment, but very few (0.03%) were differentially altered between Polµ^−/−^and wild-type mice (Figure S10B, C). This group of genes includes *Igfbp3, Lrrc46, Erdr1, Myo1g, Tmed4, Nipsnap1*, and *Ascc2.* Low quantitative differences were confirmed for functions (0.8- and 1.3-fold, for Igfbp3 and Erdr1 respectively with a plausible impact; no obvious relationship could be established with the other modulated genes, and no other major player in oxidative stress control was revealed as being involved (Figures S9A and S10). All together, the results suggest a moderately increased damage-resistance state in the Polµ^−/−^ mice. Additionally, we analyzed expression of selected genes involved in brain development or homeostasis (Figure S10B), in response to an acute oxidative stress challenge. Only *Bmp4*, clearly induced after paraquat treatment in wild-type animals, was down-regulated (0.6-fold) in Polµ^−/−^ mice. Based on previously published evidences [35], [36], [37], the differences in *Bmp4* up-regulation upon acute oxidative stress challenge (Figure S9B) could imply that Polµ^−/−^ brain could be less prompted to suffer premature senescence associated with Bmp4 enhanced signaling [37].

### The Brain of Polµ^−/−^ Mice Presents an Altered Endocrine Network

**Figure 6:**
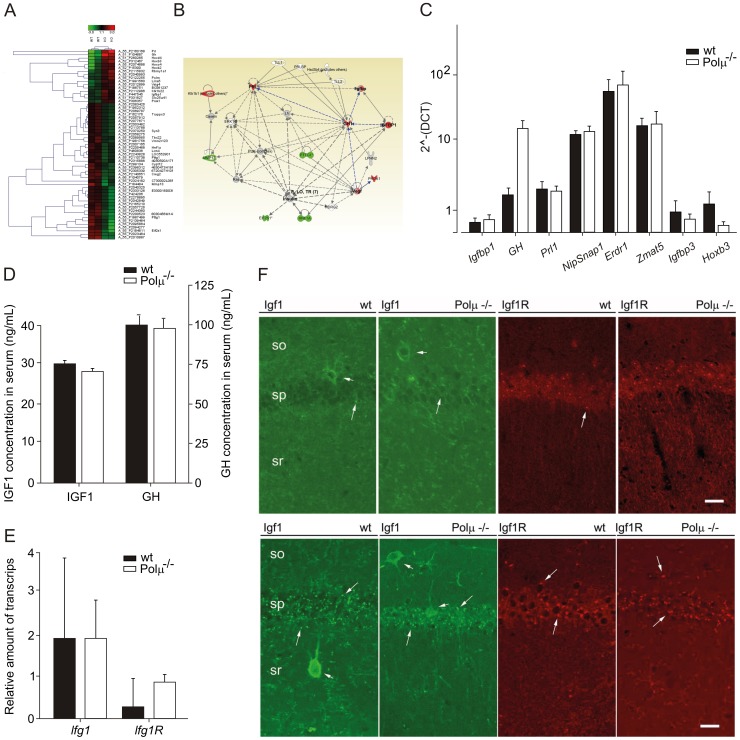
Polµ^−/−^ mice brain presents an altered endocrine network. (**A**) Hierarchical clustering of the differential mRNA expression between wild-type and Polµ^−/−^ old (18 months) mice. (**B**) Schematic representation of the expression network detected using the software Ingenuity. (**C**) qRT-PCR analysis of genes belonging to the previously described network (**B**) and others found in DNA expression arrays carried out with brain of acutely treated animals (Figure S10). Black bars denote values for wild-type animals and white bars correspond to Polµ^−/−^ animals. (**D**) Circulating levels of IGF1 and GH (ng/mL) were monitored by ELISA in old (21–24 months) animals. (**E, F**) qRT-PCR expression (**E**) and immunoconfocal analysis (**F**) of Igf1 and Igf1R in the hippocampal CA1area from wild-type and Polμ^−/−^ young adult (4 months) mice. Igf1 immunoreactivity-labeled non-pyramidal cells (short arrows) of *stratum oriens* (so), *stratum pyramidale* (sp), and *stratum radiatum* (sr; not shown); no differences in labeling frequency or labeling intensity were found between Polμ^−/−^ and wild-type mice. Igf1R-immunostaining delineated cell processes in the stratum radiatum (sr) of Polμ^−/−^ but not of wild-type mice. As compared with the case of aged mice, unspecific punctate labeling (long arrows) was infrequent in these tissue sections. Slight differences were found, however, in the occurrence of Igf1-immunoreactive cells in the CA1 area of aged mice, and in the expression of Igf1R between young wild-type and Polμ^−/−^ mice. Scale bar, 25 µm.

In an attempt to obtain further clues of the relaying mechanism, we carried out DNA expression array analysis of brain samples in both adult (8–11 months) and old (18 months) animals. Comparative expression profiles between 18-month-old Polµ^−/−^ and wild-type mice demonstrated a very similar pattern and small quantitative differences (Figure 6A). Bioinformatics analysis revealed two gene networks altered in the Polµ^−/−^ mice. The first gene network (Figure S10) altered in the old Polµ^−/−^ mice pivots around several Hox genes (*A3, B2, B3, B4,* and *C4*), with no obvious relationships with brain functions recognized. The second is centered on endocrine functional differences, and is mainly based on the brain modulation of *GH, Igfbp1, Prl1, Pttg1,* and *Prok1* (Figure 6B). Several, but not all (there was an extremely high inter-animal variability), of the revealed genes were confirmed by qRT-PCR (Figure 6C). The fact that paired-age (18–24 w) Polµ^−/−^ mice, in comparison with wild-type animals, do not present significant differences in serum concentration (Figure 6D) of either GH (91.29±8.03 ng/mL vs. 94.17±10.59 ng/mL, respectively) or IGF1 (28.96±3.54 ng/mL vs. 26.60±2.59 ng/mL, respectively) strongly suggests that the potential derived defects must be local.

Because the neuroendocrine network detected is centered on the regulation of *GH/Igf1* and it has been demonstrated that the simple heterozygous inactivation of brain insulin-like growth factor receptor (*Igf1R*) led to a consistent somatotropic deficit (without any detectable effects on other brain functions) and increased mean lifespan [38], we evaluated whether lack of Polµ could affect Igf1R expression. Real-time reverse transcription PCR expression analyses in old mice revealed no significant differences in Igf1 expression or up-regulation of *Igf1R* (Figure 6E). No significant differences were detected in the expression level or localization of Igf1R immunoreactivity in the CA1 area of old Polµ^−/−^ brain (Figures 6F and S9). However, IGF1 immunofluorescence suggested an increased expression of the protein in CA1 non-pyramidal cells of aged Polµ^−/−^ brain (higher numbers of Igf1+ cells and with increased expression). In contrast, no changes in CA1 IGF1 immunostaining were observed in 4-month-old Polµ^−/−^ brain. Therefore a more intense IGF1 immunostaining of non-pyramidal cell processes in Polµ^−/−^ brain could indicate an increased level of Igf1 expression in that neuron population (Figure 6F). Altogether, these results and the down-regulation of *Igfbp3* (Figure 6C) do not support an important involvement of the classical somatotropic axis in the better preservation of cognitive functions in the old Polµ^−/−^ mice.

## Discussion

Trace conditioning is a hippocampus-related paradigm of associative learning [18], [19]. Eyeblink conditioning evokes a change in strength at the hippocampal CA3-CA1 synapse in behaving mice [17] and evaluates associative learning capacity of them mouse model under study. In addition, LTP is a well-known form of synaptic plasticity that shares many properties with the synaptic potentiation evoked by motor or cognitive learning [17], [21]–[25]. Comparative analysis of 18-month-old wid-type and Polµ^−/−^ mice demonstrated an improved synaptic plasticity Polµ^−/−^ mice during associative learning, and LTP evoked in alert behaving mice confirmed that old mice deficient in Polµ demonstrate a better preservation of these two high level brain functions.

Analyses of young/adult (3–8 months) Polµ^−/−^ mice for some parameters (Figure S2) showed that animals are less active then wild-type controls, whereas at old–ages (18 months) the differences get inverted. We confirmed a role for Polµ in early B-cell development [10] and recently it has been confirmed that retina development is delayed Polµ^−/−^ mice (E. de la Rosa et al., to be published). So, in this context it is not difficult to assume that the poorer results obtained in some tests in young animals originates from a moderate weak impairment during central nerve system development derived from deficit in DSB repair. In fact it is possible that the phenotypes observed in the adult/old animals might be conditioned from a compensatory re-equilibrium of coordinated pathways during development, as has been recently proposed for some functions that affect mitochondrial aging [38]. In any case, what is it more surprising is that even with this apparent initial deficit, the adult neural system seems to cope perfectly well along the whole life, being apparently better preserved along physiological aging.

Physiological aging has a clear effect on LTP induction [39], which was significantly smaller in both groups of 18-month-old mice with respect to 3-month-old matched genotypes (Figure 3). Therefore we have tried to define whether an altered aging could be contributing to the improved brain maintenance in old Polµ^−/−^ mice. Immunohistochemical studies suggested that the enhanced functional state of the CA3–CA1 synapses was not related to an enriched axonal projection to CA3 pyramidal neurons. The present histological and immunohistochemical studies suggested that the enhanced functional state of the hippocampal circuits in aged Polµ^−/−^ as compared to aged wild-type mice did not correlate with an improved maintenance of neuronal circuits in the hippocampus along the protracted aging observed in these mice. This preliminary conclusion requires refinement, since more subtle changes in neuronal circuitry in aged Polµ^−/−^ mice cannot be excluded.

At the cellular and molecular levels several pathways involved in modulation of aging were studied, and potential compensatory effects by the closest PolX DSB repair enzymes, were ruled out. Brain of Polµ^−/−^ mice does not demonstrate a better maintenance of telomeres, incremented autophagy or significant reduced DSB. Based on the main proposed role for Polµ *in vivo* [11], [12], [15] we analyzed mutagenic bypass repair activity (8oxoG:dAMP) on an artificial 8oxoG modeled lesion, in crude brain extracts. Evaluation of wild-type versus Polµ^−/−^ brain extracts from old mice demonstrated a significant difference for the generation of full-length “bypassed/ligated” products, especially prominent when dATP (error-prone reaction) was provided in comparison with dCTP (error-free reaction), in wild-type extracts. These results, in companion of the parallel control reactions, imply that brains from old wild-type animals have a significant mutagenic bypass activity in templates harboring 8oxoG lesions, as the most representative oxidative damage, and that this mutagenic potential is significantly reduced in the absence of Polμ. Although a direct association between this global reduction in mutagenic bypass of 8oxoG lesions evaluated in crude brain extracts, mainly contributed by glial cells, and the positive effects demonstrated in the preservation of high level brain functions could be difficult to be solidly established, it can be envisioned as a relevant difference that could impact in brain physiology maintenance, directly or indirectly. This evidences, combined with a more efficient mitochondrial activity (Figure 5) and the reported reduced reactive oxygen species (ROS) levels in Polµ^−/−^ mice [15], could constitute a less aggressive environment, both for the genetic and non-genetic constituents of the cell.

Due to the established association between a defective or mutagenic DNA repair capacity, oxidative stress and the aging process, and the unexpectedly improved brain function in 18-month-old Polµ^−/−^ mice, we hypothesized that brain aging could be delayed in Polµ^−/−^ mice. Analysis of a panel of candidate genes showed that one third of them (*Egln3*, *Bid*, *Acin1*, *C1qa*, *Mrps12,* and *Igf1*) were reduced in Polµ^−/−^ brain, and the evolution of the expression levels of *C1qa and Bid* demonstrate a pattern compatible with a putative retardation in brain aging in Polµ^−/−^ brains. However, other previously proposed potential aging markers in CNS were not found to be significantly modified or even up-regulated, such as P16 (Text S1 and Figure S9A) in Polµ^−/−^ old brains. Further global comparative DNA array expression analysis of brain samples in both adult (8–11 months) and old (18 months) animals confirmed small differences between old Polµ^−/−^ and wild-type brains, focusing the attention in two networks. The first is structured around several Hox genes (Figure S10), but with no recognized implications in brain function or aging. Clarification of their putative involvement in the better preservation of high-level brain functions in Polµ^−/−^ mice will require a dedicated and intense effort. The second network is centered on endocrine functional differences, and is mainly based on the brain modulation of *GH, Igfbp1, Prl, Pttg1,* and *Prok1;* some of the genes involved have been associated with aging. PRL seems to be markedly increased in serum along aging (hyperprolactinemia), GH secretion declines during normal aging, resulting in lower serum levels of IGF1, and very disparate results have been reported with regard to Igbp-1 [40]. In the same sense, it has been reported that chronic treatment with recombinant GH mitigates age-related cognitive decline, enhancing basal synaptic transmission and both AmpaR-dependent basal synaptic transmission and LTP [41], [42].

However evaluation of circulating levels of GH and IGF1 in paired-age old wild-type and Polµ^−/−^ mice did not reveal significant differences, suggesting that, if relevant, those differences might be local. In this sense, it was demonstrated that the simple heterozygous inactivation of brain insulin-like growth factor receptor (*Igf1R*) led to a consistent somatotropic deficit and increased mean lifespan [38]. In this context, we confirmed that lack of Polµ does not affect neither *Igf1* expression nor up-regulation of *Igf1R* (Figure 6E) in old mice. However, IGF1 immunofluorescence suggested an increased expression of the protein in CA1 non-pyramidal cells of aged Polµ^−/−^ brain, which is not evident in young (4-month-old) animals. Altogether, these results and the down-regulation of *Igfbp3* (Figure 6C) do not support an important involvement of the classical somatotropic axis in the better preservation of cognitive functions in the old Polµ^−/−^ mice, although the local overexpression of *Igf1/GH* by some subgroups of cells could play some role.

Finally, Polµ^−/−^ and wild-type adult mice were treated with an acute dose of paraquat and brains analyzed as an experimental model to evaluate the putative involvement of a differential oxidative stress response in the described phenotype. DNA expression array analysis demonstrated few (0.03%) but consistent differences between Polµ^−/−^and wild-type mice. Main genes revealed include *Igfbp3, Lrrc46, Erdr1, Myo1g, Tmed4, NipSnap1*, and *Ascc2. Igfbp3* has previously been demonstrated to be up-regulated in several cell systems as a consequence of oxidative stress, mediating in amplification of hyperglycemic damage [43]; *Igfbp3* is down-regulated (0.8-fold) in the resting brain of Polµ^−/−^ mice. *Erdr1* (erythroid differentiation regulator 1) was moderately up-regulated (1.3-fold) in non-treated (basal) animals (Figure 6C). Erdr1 has been defined as a pro-apoptotic or anti-metastatic factor [44], but also as a nuclear factor (HoxB4-like) able to promote self-renewal hematopoietic stem cells (HSCs) after forced overexpression [45]. Therefore, it is tempting to speculate that the moderate increase demonstrated in Polµ^−/−^ mice could contribute to a better maintenance of tissue homeostasis. No obvious relationship could be established with the other modulated genes, and no other major player in oxidative stress control was revealed involved (Figures S9A and S10). Additionally, analysis of more candidate genes involved in brain development or homeostasis (Figure S10B) revealed *Bmp4* as down-regulated (0.6-fold) in Polµ^−/−^ mice after paraquat treatment. Bmp4 is a multifunctional growth factor with pleiotropic roles whose enhanced signaling has negative implications for adult hippocampal neurogenesis [35]. Conversely, a down-regulation of *Bmp4* mediates the positive effect of elective exercise on hippocampal neurogenesis and cognition [36]. The differences in *Bmp4* up-regulation upon acute oxidative stress challenge (Figure S9B) suggest that Polµ^−/−^ brain could be less prompted to suffer premature senescence associated with Bmp4 enhanced signaling [37]. All together, these results strongly suggest that Polµ deficiency favors an aging slow-down of some brain functions, being probably mediated by a probable enhanced damage-resistance, although a formal demonstration is needed. This interpretation is in full agreement with the recently demonstrated lifespan extension phenotype in Polµ^−/−^ mice (Escudero et al., submitted).

Genetic ablation of other members of the NHEJ pathway (*Ku70*, *Ku80*, *DNA-PK_CS_*, *XRCC4*, or *Lg IV*) generates a severe DNA repair deficiency, promoting global premature replicative senescence and B and T immunodeficiency [2]. Particularly interesting is the phenotype described for the *Ku80* knockout mice, which exhibit a premature aging, associated with an accelerated organismal senescence, but a lower incidence of tumors (about 10-fold lower) with an earlier onset [46]. Paradoxically, it was demonstrated that *Ku80* knockout mice present a significant decrease in basal somatic point mutation (a reduction of around 2-fold) in both liver and brain [46], and an almost complete absence of chromosomal rearrangements (more than 30-fold). These unexpected findings were interpreted as formal proof for the *in vivo* co-operation between NHEJ and HR pathways, rendering ― in the *K80* knockout context ― a more accurate DSB repair due to the augmented participation of HR instead of the intrinsically mutagenic DNA repair mediated by the NHEJ pathway [46]. Following the hypothesis established in *Ku80* knockout mice, the fact that Polµ^−/−^ mice display a milder DNA repair deficiency [15] could conform a more favorable scenario for evidencing the co-operation between NHEJ and HR pathways. Preliminary independent studies (Escudero et al., submitted) have obtained compatible results with several cellular lineages with reduced Polµ activity, thereby outlining a plausible molecular mechanism for the delayed brain-aging phenotype, generated in origin by a less efficient but more conservative global DNA repair status of neural cell lineages.

### Concluding Remarks

Here we have described how mice genetically deficient in Polµ, a novel member of the NHEJ pathway, show a significantly better maintenance of cognitive/learning abilities and activity-dependent synaptic plasticity in hippocampal circuits during aging. This phenotype is probably due to delayed brain aging. The current molecular models for age-related deterioration point to the combined accumulation of multiple, minute changes in the regulation of genes and pathways as the most plausible cause of decline in cellular functions. Following the compatible working model hypothesis for Polµ function *in vivo*, we are tempted to speculate that the absence of Polμ function, which is prone to non-conservative end-joining repair, could provoke a less efficient but more conservative NHEJ repair, affecting mitochondrial biological efficiency and maintaining a lower chronic rate of ROS generation. The global physiological cell status would delay the typical organismal evolution that accompanies aging.

In summary, DNA polymerase µ activity influences brain aging. Polµ^−/−^ mice demonstrate a delayed aging, supported on a reduced error-prone DNA oxidative repair activity and a more efficient mitochondrial function.

## Materials and Methods

### Mice

The generation of Polµ^−/−^ mice and their wild-type counterparts has been described previously [15]. Mice were bred in our specific pathogen-free facility, and were routinely screened for pathogens, at the Animal House of the Centro Nacional de Investigaciones Cardiovasculares (CNIC, Madrid, Spain). Electrophysiological and behavioral experiments were performed, at the Animal House of the Pablo de Olavide University (Universidad Pablo de Olavide, Sevilla, Spain), with young (3-month-old), medium-age (8-month-old), and aged (18-month-old) male homozygous Polµ^−/−^ mice and their corresponding littermate controls. When indicated wild-type and Polµ^−/−^ mice (8–12 w) where injected with paraquat (50 mg/kg; i.p.), animals were sacrificed 7 h later and brains and liver extracted for further analyses. All experiments were performed in accordance to the guidelines of the European Union Council (86/609/EU) and current Spanish regulations (BOE 252/34367-91, 2005) for the use of laboratory animals in chronic studies. Experiments were also approved by the local institutional Ethics Committee for animal care and handling.

### Locomotion Test

Locomotion was measured in an activity box (26×39 cm; Cibertec, Madrid, Spain) as indicated by the number of broken light beams during periods of 10 min. Measurements of locomotor activity were carried out in a total of 15 mice/group of 3-, 8-, and 18-month-old wild-type and Polµ^−/−^ animals.

### Rota-rod Test

The rota-rod test is a behavioral task assessing motor coordination performance. In this study, we used an accelerating rota-rod treadmill (Ugo Basile, Varese, Italy). Mice (n = 15 per group) were placed on the rod and tested at 25 rpm, for a maximum of 400 s at each speed. Between trials, mice were allowed to recover in their home cages. The total time that each animal was able to stay on the rod was computed as the latency to fall, recorded automatically by a trip switch under the floor of each rotating drum. Mice were tested for 4 consecutive days, and the results averaged to obtain a single value for each group at the selected rotational speed [47].

### Surgical Preparation of the Experimental Animals

Before surgery, animals were housed in collective cages (n = 5 per cage). Mice were kept on a 12 h light/dark cycle with constant ambient temperature (21±1°C) and humidity (50±7%). Food and water were available *ad libitum*. For classical conditioning of eyelid responses, a total of 10 successful animals for each experimental group were used. LTP studies were carried out on 10 additional animals per group. Mice were anesthetized with 0.8–3% halothane (AstraZeneca, Madrid, Spain) and placed in a stereotaxic apparatus (David Kopf Instruments, Tujunda, CA, USA). Halothane was administered through a home-made mask from a calibrated Fluotec 5 (Fluotec-Ohmeda, Tewksbury, MA, USA) vaporizer, at a flow rate of 1–4 L/min oxygen. Once anesthetized, animals were implanted with bipolar stimulating electrodes on the left supraorbital nerve and with bipolar recording electrodes in the ipsilateral orbicularis oculi muscle [17]. Electrodes were made of 50 µm, Teflon-coated, annealed stainless steel wire (A-M Systems, Carlsborg, WA, USA), with their tips bared for ≈0.5 mm. Electrode tips were bent as a hook to facilitate a stable insertion in the upper eyelid. Animals were also implanted with bipolar stimulating electrodes aimed at the right (contralateral) Schaffer collateral-commissural pathway of the dorsal hippocampus. The selected implantation site was located 2 mm lateral and 1.5 mm posterior to Bregma, and 1.0–1.5 mm from the brain surface [48]. A recording electrode aimed at the ipsilateral *stratum radiatum* underneath the CA1 area (1.2 mm lateral and 2.2 mm posterior to Bregma; depth from the brain surface, 1.0–1.5 mm; ipsilateral to the stimulating ones) was also implanted. Stimulating and recording electrodes were made of 50 µm, Teflon-coated tungsten wire (Advent Research Materials Ltd., Eynsham, England). The final position of hippocampal stimulating and recording electrodes was determined following the recording procedures explained below [17]. A 0.1 mm bare silver wire was affixed to the skull as ground. All the implanted wires were connected to two four-pin sockets (RS-Amidata, Madrid, Spain). Sockets were fixed to the skull with the help of two small screws and dental cement [17], [49]. After surgery, animals were housed in individual cages. Two weeks were allowed for animal recovery before the start of classical conditioning or LTP experiments.

### Classical Conditioning Procedures

For classical conditioning of eyelid responses, the animal was placed in a small (5×5×10 cm) plastic chamber located inside a larger Faraday box (30×30×20 cm). Conditioning was achieved using a trace paradigm consisting of a tone (20 ms, 2.4 kHz, 85 dB) presented as a conditioned stimulus (CS), and a cathodal, square pulse applied to the supraorbital nerve (500 µs, 3×threshold) as the unconditioned stimulus (US). The US started 500 ms after the end of the CS. A total of four habituation, 10 conditioning, and five extinction sessions were carried out for each animal. A conditioning session consisted of 60 CS-US presentations, and lasted ∼30 min. For a proper visual identification and further analysis of conditioned responses (CRs), the CS was presented alone in 10% of the cases. CS-US paired presentations were separated at random by 30±5 s. For habituation and extinction sessions, only the CS was presented, also for 60 times per session, at intervals of 30±5 s. Eyelid responses were determined by recording the electromyographic (EMG) activity of the orbicularis oculi muscle ipsilateral to US presentation.

### Hippocampal Recording and Stimulation Procedures

Electromyographic (EMG) recordings were carried out with the help of Grass P511 differential amplifiers within a bandwidth of 0.1 Hz–10 kHz (Grass-Telefactor, West Warwick, RI, USA). Hippocampal recordings were made with a high impedance probe (2×10^12^ Ω, 10 pF).

As a criterion, we accepted as a CR the presence, within the CS-US interval, of EMG activity of the orbicularis oculi muscle lasting >10 ms and initiated >50 ms following CS onset. Moreover, the integrated EMG activity recorded during the CS-US period had to be at least 2.5 times greater than the averaged activity recorded immediately before CS presentation [50].

Electrodes were surgically implanted in the CA1 area, using as a guide the field potential depth profile evoked by paired (10–500 ms interval) pulses presented at the ipsilateral Schaffer collateral pathway. The recording electrode was fixed at the site where a reliable monosynaptic field excitatory postsynaptic potential (fEPSP) was recorded. Synaptic field potentials in the CA1 area were evoked during habituation, conditioning, and extinction sessions by a single 100 µs, square, biphasic (negative-positive) pulse applied to Schaffer collaterals 300 ms after CS presentation. Stimulus intensities ranged from 50 to 350 µA. For each animal, the stimulus intensity was set well below the threshold for evoking a population spike ― usually at 30–40% of the intensity necessary for evoking a maximum fEPSP response [48]. An additional criterion for selecting stimulus intensity was that a second stimulus, presented 40 ms after a conditioning pulse, evoked a larger (>20%) synaptic field potential than the first one [17], [51]–[52].

Field EPSP baseline values were collected 15 min prior to LTP induction. For this, we presented single (100 µs, square, biphasic) pulses at a rate of 3/min. For evoking LTP, each animal was presented with five 200 Hz, 100 ms trains of pulses at a rate of 1/s. This HFS protocol was presented 6 times in total, at intervals of 1 min. The 100 µs, square, biphasic pulses used to evoke LTP were applied at the same intensity used for baseline recordings. After HFS, single pulses were presented again for 120 min at the same rate as for baseline records. Recordings were repeated 24 h later for 15 additional minutes.

### Identification of the Implanted Sites

At the end of behavioral experiments, mice were deeply re-anesthetized (sodium pentobarbital, 50 mg/kg), and perfused transcardially with saline and 4% phosphate-buffered paraformaldehyde. Selected sections (50 µm) including the dorsal hippocampus were mounted on gelatinized glass slides and stained using the Nissl technique with 0.1% Toluidine blue, to determine the location of stimulating and recording electrodes [17].

### Electrophysiology Data Analysis

EMG and hippocampal activity, and 1-volt rectangular pulses corresponding to CS and US presentations, were stored digitally on a computer through an analog/digital converter (CED 1401 Plus, Cambridge, England), at a sampling frequency of 11–22 kHz and with an amplitude resolution of 12 bits. Commercial computer programs (Spike 2 and SIGAVG from CED) were modified to represent EMG, EEG, and extracellular synaptic field potential recordings. Data were analyzed off-line for quantification of CRs and of fEPSP slopes with the help of home-made representation programs [17],[50]–[53]. Computed results were processed for statistical analysis using the SPSS for Windows package. Unless otherwise indicated, data are represented as the mean ± SEM. Acquired data were analyzed using a one- and two-way ANOVA test, with session and group as repeated measure. Data collected from the behavioral tests (actimeter and rota-rod) were also analyzed with one- and two-way ANOVA. Contrast analysis was added to study further significant differences. Regression analysis was used to study the relationship between fEPSP slopes and the percentage of CRs.

### Microarray Gene Expression Profiling

#### Sample labeling and microarray hybridization

The one-color Microarray-Based Gene Expression Analysis Protocol (Agilent Technologies, Palo Alto, CA, USA) was used to amplify and label RNA. Briefly, 400 ng of total RNA was reverse transcribed using T7 promoter Primer and MMLV-RT. cDNA was then converted to aRNA using T7 RNA polymerase, which simultaneously amplifies target material and incorporates cyanine 3-labeled CTP. Cy3 labeled aRNA (1.65 µg) was hybridized to a Whole Human Genome Microarray 4×44 K (G4112F, Agilent Technologies) for 17 hours at 65°C in 1X GEx Hybridization Buffer HI-RPM in a hybridization oven (G2545A, Agilent Technologies) set to 10 rpm. Arrays were washed according to the manufacturer’s instructions, dried by centrifugation, and scanned at 5 mm resolution on an Agilent DNA Microarray Scanner (G2565BA, Agilent Technologies) with the default settings for 4×44 K format one-color arrays. Scanned images were analyzed with Feature Extraction software (Agilent Technologies).

#### Data analysis

Feature Extraction data files were imported into GeneSpring® GX software version 9.0 (Agilent Technologies). Quantile normalization was performed and expression values (log2 transformed) were obtained for each probe. Probes were also flagged (*Present*, *Marginal*, *Absent*) using GeneSpring® default settings. Probes with signal values above the lower percentile (20^th^), and flagged as *Present* or *Marginal* in 100% of replicates in at least one out of the two conditions under study, were selected for further analysis. Data were edited and analyzed in R (R Development Core Team) using different packages of the Bioconductor project [54], as well as custom written R routines.

#### Data processing

Data were read into R and processed using the *Agi4×44PreProcess* Bioconductor package as follows. *Agi4×44PreProcess* options were set to use the *MeanSignal* and the *BGMedianSignal* as foreground and background signals, respectively. Then, data were background corrected and normalized between arrays using the *half* and *quantile* methods. The *half* method produces a positive background corrected signal by subtracting the background signal from the foreground signal keeping any intensity less than 0.5 equal to 0.5 to produce positive corrected intensities. Then data were normalized between arrays using the *quantile* method [55]. A constant equals 50 was added to the intensities before the log transformation in order to reduce the signal variability of the low intensity expressed genes. The AFE image analysis software attaches to each feature a set of flags that identify different quantification properties of the signal. *Agi4×44PreProcess* uses these flags to filter out features that 1) are controls 2) are out of the dynamic range of the scanner and 3) are outliers. To keep features within the dynamic range 3 independent levels of filtering can be done to ensure that 1) the signal is distinguishable from the background, 2) the signal is found, and 3) the signal is not saturated. For each of these filtering steps, we required for each feature that more than the 75% of its replicates within at least one experimental condition have a quantification flag denoting that the signal is within the dynamic range. In addition, for each replicated feature across the whole set of samples, we filtered out those probes that had more than 25% of its replicates in at least one experimental condition with a flag indicating presence of outliers. After the completion of all the pre-processing steps there were 26,670 (T20 vs. T3 data set) features available for the statistical analysis. Finally, *Agi4×44PreProcess* maps each Agilent manufacture’s probe identifier to its corresponding accession number, gene symbol, gene description and Gene Ontology identifiers (GO; The Gene Ontology Consortium) using the Bioconductor annotation *hgug4112a.db.* Data were finally analyzed using the Ingenuity Systems IPA software (Ingenuity Systems Inc., CA, USA).

### Statistical Analysis

The differential expression analysis was done using the linear modeling features implemented in the Bioconductor *limma* package. The *limma* package incorporates empirical Bayes methods to obtain moderated statistic [56]. To estimate the differential expression between the different experimental conditions in the two data sets (T20 vs. T3 and P21 vs. P2), the following linear model was fitted to each gene: 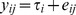 where 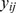 is the observation of treatment i^th^ for individual j^th^, 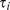 is the effect of the i^th^ treatment and 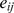 is the experimental error, assumed normally distributed with 0 mean and variance 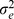. The differentially expressed genes due to differences in the treatments were discovered by testing for each gene the hypothesis of no differences between gene signals under different treatment using the estimates 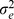.

To reduce the number of genes for the multiple testing correction without missing relevant information, a non-specific filtering was done by removing the genes that showed either a constant expression between samples (IQR <0.50) or low expression signal (log2 expression <5 in all samples) using the Bioconductor *genefilter* function. Since the *eBayes* function estimates the average variability of the genes on the microarray, the non-specific filtering was done after the *eBayes* correction. Then, the multiple comparisons of genes were taken into account by controlling the false discovery rate, which was estimated in terms of the q value statistic using the Bioconductor *qvalue* package [57].

To integrate significant expression profiles into functional categories we performed a Gene Ontology (GO)-based statistical analysis using the *hyperGTest* function of the *GOstats* package [58]. The *hyperGTest* computes hypergeometric p values to test for overrepresentation and underrepresentation of each GO term in a given subset of genes in comparison to the distribution of GO terms in a defined universe of genes. The universe included those genes that were above the background signal and had a known GO term in the corresponding database. Duplicated genes were removed before the GO analysis.

### Quantitative Real Time RT-PCR Evaluation

Total RNA was isolated from brain of young (2 months old), and old (11, 14, and 19 months old) mice using TRI® REAGENT (Sigma, P/N: T9424) as indicated by the manufacturer. cDNA synthesis was performed using a Reverse Transcription kit (Promega, P/N: A3500) following the manufacturer’s protocol. Gene expression was evaluated by Real Time PCR or TaqMan assay and expressed as mRNA level normalized to a standard housekeeping gene (36B4 and β-actin, respectively). Real-time Quantitative PCR assays were performed using Applied Biosystems 7000 Sequence Detecting system, and Power SYBER Green PCR Master Mix (Applied Biosystems, P/N:4367659) or TaqMan 2X PCR master mix (Applied Biosystems, P/N: KP0054). Ten ng of cDNA per animal brain was used to perform triplicate PCR analyses per experiment. We used the primers summarized in Table S1 for RT-PCR amplification. *Cox2* cDNA was amplified by TaqMan, with the probe 5′-TCATGAGCAGTCCCCTCCCTAGGACTTAA-3′; (5′ FAM 3′ TAMRA), the upstream primer 5′-TTTCATCTGAAGACGTCCTCCA-3′ and the downstream primer 5′- GGCCTGGGATGGCATCA-3′. The rest of the cDNAs were amplified using the corresponding TaqMan gene assay, designed and provided by Applied Biosystems. These were the following: Mm00432142_m1 for *C1qa*, Mm00432448_m1 for *P21*, Mm00480750_m1 for *Perp*, Mm00477210_m1 for *Pten*, Mm00472200_m1 for *Egln3*, Mm00432073_m1 for *Bid*, Mm000479895_m1 for *Acin1*, Mm00546086_m1 for *Gfap*, Mm00431960_m1 for *Atp5a1*, Mm00465919_m1 for *Mrpl28*, Mm00488728_m1 for *Mrps12*, Mm00809812_m1 for *Timm17a*, Mm00600325_m1 for *Ndufa10*, Mm00802841_m1 for *Igf1R*, Mm00439560_m1 for *Igf1*, and 4352933-E for *β-Actin*. The amount of transcripts was calculated as follows: 2∧(–Δct), where Δct is the ct value normalized to the corresponding housekeeping gene.

### Immunoconfocal Analysis

Animals for immunohistochemistry (n = 19) were deeply anesthetized with ketamine-xylazine, i.p., and perfused transcardially with saline followed by 4% parafomaldehyde in 0.12 M phosphate buffer, pH 7.2. Brains were dissected out, postfixed overnight at 4°C, and stored in PBS or in an ethylene glycol-glycerol-PBS anti-freeze solution at 4°C. Cryoprotected blocks were sectioned at 50 µm in a freezing sliding microtome. Sections were blocked in 4% bovine serum albumin, 3% normal horse serum, 0.1% Triton X-100, and 0.05% azide in PBS for 2 h. Then, sections were incubated in the primary antibodies diluted in the same blocking solution overnight. Primary antibodies used were mouse monoclonal antibody M23 to IGF1 (Abcam, Cambridge, UK; 1∶300), mouse monoclonal antibody AP-20 to microtubule-associated protein 2 (map2, 2a+2b; Sigma; 1∶250), rabbit polyclonal antibody sc-713 to IGF1Rβ (Santa Cruz Biotechnology, CA, USA; 1∶150) and rabbit polyclonal antibody to the Zn^2+^ vesicular transporter ZnT3 (Synaptic Systems, Göttingen, Germany; 1∶500). Secondary antibodies were Alexa Fluor 488 anti-mouse IgG and Alexa Fluor 546 anti-rabbit IgG (both from Molecular Probes - Invitrogen, Barcelona, Spain; 1∶500). The secondary antibodies were diluted in blocking solution and applied for 3 h. Sections were then washed in PBS and coverslipped with Citifluor (London, UK). Sequential confocal images were obtained in a Leica TCS SL confocal microscope.

For the 53BP1 immunofluorescence, paraffin sections were deparaffined, rehydrated and unmasked with Trilogy pretreatment solution (CELL MARQUE, 20X), by incubation in 1X of this during 15 min in a microwave at full power. Paraffin sections were washed two times in TBS containing 0.2% Tween (TBST) 5 min each one, and blocked (10 min) in CAS-Block (Invitrogen). Preparation were incubated 1h with anti 53BP1 antibody (NovusBio) diluted 1/50 in CAS-Block (Invitrogen). After washing with TBST and incubated with the secondary antibody (Anti-Rabbit 568) during 45 min, slides were washed again for two times with TBST and mounted with DAPI (VectaShield mounting medium, Vector). Confocal images were acquired on Leica SPE microscope using LAS AF Software. Tiff images were analyzed with Images J program, and were scored the number of 53BP1 positive nuclei.

### Silver Staining of Axonal Systems in the Hippocampus

Adult brains (n = 10) perfused as above were sectioned at 50 µm in a Vibratome, mounted on gelatinized slices and silver stained as described elsewhere [59].

### Telomere Length Evaluation

DNA was extracted from brain tissue through the DNAzol methods adaptation. Average telomere length was measured from total genomic DNA using a real time quantitative PCR method. PCR reaction was performed on the Mastercycler realplex2 (Eppendorf), using telomeric primers; primers for the reference control genes were mouse single copy gene 36B4 [60], and human single copy gene (retinoic X receptor b gene) [61]. Each reaction in the assay was performed as references above. Equal amounts of genomic DNA (100 ng) were used with several duplicates. The reaction condition in the thermocycler includes first step at 95°C for 10 min followed by 40 cycles of 95°C for 15s and 60°C anneal-extend step for 1 min. We made internal standard curve with the relative telomere length ratio (T/S) of human cells lines of which the telomeric length is known such as Tin2, LXSN and Tin 2–13 [62], to estimate the telomeric length of our samples. Ratio (T/S) is defined as 2∧-(delta Ct), where delta Ct =  mean Ct telomere/mean Ct single copy internal gene.

Quantitiative FISH was carried out using a Cy3- labeled LL(CCCTAA)3 peptide nucleic acid (PNA) telomeric probe (Eurogentec, Liège) as previously described [59] with the following modifications. After hybridization slides were washed three times with PBS-0.1% Tween for 10 min at 60°C and dehydrated through an ethanol series (70%, 90% and 100%; 5 min each). Slides were then counterstained and mounted in Vectashield H-1200 mounting medium. Digital images were acquired as described above. Telomere signals were captured with the same exposure time in all samples. Telomere length (in kb) was extrapolated from the fluorescence of hTert-immortalized 82-6 fibroblasts expressing either TIN2 or TIN2-13 proteins with known and stable telomere lengths (3.4 and 8.4 kb respectively; kindly provided by Dr Judith Campisi). Telomere signals from at least 20–30 nuclei per group were quantified using TFL-Telo (version 2), kindly provided by Dr Peter Lansdorp (British Columbia Cancer Centre, Vancouver, Canada). All images were captured and analyzed in parallel on the same day by an experimenter blinded to the treatment groups.

### Evaluation of Mitochondria Activity

Preparation of mitochondrial fractions and spectrophotometric activities of individual complexes were performed as described elsewhere [63]. Evaluation of the different parameters of mitochondrial activity was carried essentially as previously described [64] and mitochondrial supercomplex study also as described elsewhere [65].

### Evaluation of Mutagenic DNA Repair Activity

Extracts were prepared essentially as previously described [66]. Briefly, tissues were washed three times with ice-cold phosphate-buffered saline and resuspended in 1ml for 500 µg tissue of Buffer I (10 mm Tris-Cl, pH 7.8, and 200 mm KCl). After the addition of an equal volume of Buffer II (10 mm Tris-Cl, pH 7.8, 200 mm KCl, 2 mm EDTA, 40% glycerol, 0.2% Nonidet P-40, 2 mm dithiothreitol, 0.5 mm phenylmethylsulfonyl fluoride, 10 µg/ml aprotinin, 5 µg/ml leupeptin, 1 µg/ml pepstatin), the suspension was rocked at 4°C for 1 h and then centriguged at 16,000×g for 10 min. The supernatant was recovered and stored in small aliquots at −80°C.

PAGE-purified oligonucleotides were 5′ end labeled with [γ-^32^P]ATP by polynucleotide kinase. The oligonucleotides used to generate the DNA substrates were the following: for gapped 8OxG containing substrate, P15 (5′-CTGCAGCTGATGCGC-3′), T34^8G^ (5′- GTACCCGGGGATC.

CGTAC**8**GCGCATCAGCTGCAG-3′) and 5′ phosphate-containing D18 (5′-GTACGGATC CCCG.

GGTAC-3′), were **8** indicates the presence of an 8OxG moiety; for standard (gap-filling) polymerization assays T34^8G^ oligonucleotide was substituted for T34 (5′- GTACCCGGGG ATCCGTAC**G**GCGCATCAGCTGCAG-3′). For *ex vivo* evaluation of extracts, appropriate template primer (T/P/D) substrates were used with the clarified brain extracts (wild-type vs. Polµ^−/−^).

For standard (gap-filling or bypass mutagenic repair) polymerization assays, the incubation mixture (20 µl) contained 50 mM Tris-HCl (pH 7.5), 1 mM MnCl_2_ (alternatively 10 mM MgCl_2_), 1 mM DTT, 4% glycerol, 0.1 mg/ml BSA, 5 nM gapped DNA, the indicated concentration of NTPs, and either wild-type or Polµ^−/−^ brain extracts (10 µg). After 30 min of incubation at 30°C, reactions were stopped by addition of loading buffer (10 mM EDTA, 95% [v/v] formamide, 0.03% [w/v] bromophenol blue, 0.3% [w/v] cyanol blue) and subjected to electrophoresis in 8 M urea-containing 20% polyacrylamide sequencing gels. After electrophoresis, the unextended, degraded or extended DNA primers were detected by autoradiography, and quantified.

### Circulating Levels of GH and IGF1

Animals, at different ages, were bled (approx. 250 µl) and serum obtained and kept frozen. Circulating levels of growth hormone (GH) were determined using the Growth Hormone (GH) EIA Rat/Mouse 96 Test kit (Materlab) and IGF-1 using the IgF-1 (Rat/Mouse) EIA 96 TEST kit (Materlab), following the instruction of the manufacturer. Evaluations were done in triplicates.

## Supporting Information

Figure S1 **Mechanistic working model for** ***in vivo*** **Polµ function in NHEJ reactions. (A)** NHEJ (error-prone) and HR (error-free) are the fundamental pathways for double-strand break (DSB) repair in mammals, and both compete for the same substrates. The contribution of either mechanism is dictated by the avidity of the heterodimer ku70/ku80 and the activity of the MRX complex for the DSB (*Lee et al., 2008*), and is strictly dependent on the cell cycle status; D-NHEJ, the classical pathway dependent on DNA-PK; B-NHEJ, a novel subpathway (called backup NHEJ mechanism), recently characterized, dependent on PARP-1, DNA Ligase III, and Histone 1 (*Rosidi et al., 2008*). (**B)** The HR pathway is preferentially active in the S and G2 phases of the cell cycle when a homologous sister chromosome or chromatid is available for direct base-pairing to effect error-free repair of a DNA DSB. Conversely, the NHEJ repair pathway can be used within any phase of the cell cycle and can be error-prone. The reported comparative strength of the two mechanisms (NHEJ vs. HR) along the different phases of the cell cycle has been schematized, following recent evidence (*Kan’o et al., 2007; Mao et al., 2008; Natarajan et al., 2008*). The letter size is intended to illustrate graphically the comparative contribution of the two mechanisms in each phase of the cell cycle. (**C**) A basic model for Polµ-dependent nucleotide insertions catalyzed during DSB repair. Processed NHEJ typically results in rearrangements. End-processing activities exist to deal with terminal damage, and occasionally, incompatible ends are generated as a collateral phenomenon. Polµ is unique in that it can process incompatible overhangs: at some end sequences, Polµ action can be template-directed (error-free), but in other cases wrong nucleotides are inserted, thus contributing to mutagenesis. (**a**) Schematic representation of a minimally processed DSB (1nt 3′-protruding ends), with non-complementary sequences. (**b**) Polµ structure (in gray) depicted as 2 ellipses, the larger one representing the polymerization core, and the smaller one indicating the 8kDa domain, having an ssDNA binding cleft that contains the 5′-P binding site, critical for stabilizing enzyme/DNA binding. **c**) Synapsis/juxtaposition of the two incompatible DNA ends mediated by Polµ, in which the ends could be properly (left scheme) or wrongly (right scheme) aligned. **d**) Depending on the alignment (end-sequence dependent) and potential damage context, Polµ can insert a nucleotide complementary to the other end (green X; left column), or non-complementary (red N; central column), by virtue of its Terminal Transferase activity; additionally, and based on its high tendency to use microhomology for local primer relocalization prior to polymerization, a putative second insertion is postulated on some occasions (green X, after the red N; right column), which could facilitate ligation. (**e**) All these intermediates can be managed by DNA Ligase IV, generating different final products. (**f**) A restored wild-type sequence (Error-free; left column), or modified sequences (Error-prone), including mismatches (central column) or a +1 frameshift (right column).
(TIF)

Figure S2 **Polµ^−/−^ mice display reduced exploratory activity and enhanced sensorimotor coordination during aging. (A–B)** Motor activity (defined as number of beam interruptions per 10 min) of 3-, 8-, and 18-month-old wild-type (black bars) or Pol**µ** ^−/−^ (white bars) mice. (**C–D**) Maximum time of permanency on the rota-rod (**C**) for 3-, 8-, and 18-month-old wild-type (closed circles) or Pol**µ** ^−/−^ (open circles) mice analyzed on a rota-rod machine for 400 s per day over 5 days. (**D**) A total of n = 15 animals/group were used in these experiments. * *P*<0.05, ** *P*<0.01, two-way ANOVA.
(TIF)

Figure S3 **Characteristics of eyeblink responses evoked in young and aged Polµ^−/−^ and wild-type mice.** (**A**) A diagram indicating the location of stimulating (St.) electrodes implanted on the supraorbital nerve and electromyographic (EMG) recording electrodes implanted in the *orbicularis oculi* (O.O.) muscle. (**B**) Three superimposed records of the O.O. EMG response to the electrical stimulation of the ipsilateral supraorbital nerve collected from an 18-month-old Pol**µ** ^−/−^ mouse. Note the two short- (R1) and long- (R2) latency components characterizing the blink reflex in mammals. EMG calibration as indicated. (**C, D**) Mean (± SEM; n = 20 measurements) values collected for EMG latency (**C**) and area of rectified EMG records (**D**) of both R1 and R2 components of electrically evoked blinks in 3- and 18-month-old wild-type (black bars) and Pol**µ** ^−/−^ (white bars) mice. No significant difference (*P*≥0.425, two-way ANOVA) was observed between groups for any of the four parameters.
(TIF)

Figure S4 **Quantitative analysis of the relationships between the percentage of CRs and fEPSP slopes for the different experimental groups across habituation, conditioning, and extinction sessions.** Data collected from 3-month-old wild-type (A) and Pol**µ** ^−/−^ (B) mice and from 18-month-old wild-type (C) and Pol**µ** ^−/−^ (D) mice are illustrated. Each point represents the mean value collected from a single animal during the corresponding session. Equations corresponding to each of the three relationships included in each plot are indicated. Note that this linear regression analysis was non-significant for habituation sessions in all of the groups and for conditioning and extinction sessions as well in the 18-month-old control group.
(TIF)

Figure S5 **Paired-pulse facilitation of field excitatory postsynaptic potentials (fEPSP) recordings in the CA1 area following stimulation of the ipsilateral Schaffer collateral-commissural pathway.** Data were collected from extracellular fEPSP paired traces collected from 18-month-old wild-type and Pol**µ** ^−/−^ mice at different inter-pulse intervals. The data shown are mean ± SEM slopes of the second fEPSP expressed as a percentage of the first for the six (10, 20, 40, 100, 200, 500 ms) inter-stimulus intervals for the four experimental groups.
(TIF)

Figure S6 **Molecular characterization of Polμ−/−** **mice associated with aging. (A)** Paraffin brain sections from wild-type and Pol**µ** ^−/−^ mice (18–20 months old) were processed and stained for 53BP1 (M). (**B**) qRT-PCR expression analysis of a selection of genes (*Atg5l, Atg7l, Atg12l, Maplc3b, and Atg9a*) critical for execution or regulation of autophagy. Black bars correspond to wild-type samples and gray bars to Pol**µ** ^−/−^. (**C, D**). Telomere-length evaluation in brain tissue. In **C**, genomic DNA from wild-type or Pol**µ** ^−/−^ mice was subjected to telomere-specific PCR reaction using specific primers. The relative telomere length ratio (T/S) was calculated as indicated in Material and Methods section, and is defined as 2?-(delta Ct), where delta Ct =  mean Ct telomere/mean Ct single copy internal gene. Quantitative FISH was carried out using a Cy3- labeled LL(CCCTAA)3 peptide nucleic acid (PNA) telomeric probe as previously described (*Estrada et al., 2011*). In **D** are illustrated representative pictures of wild-type and Polμ_−/−_ samples stained for telomere length (red) and nuclei (DAPI, blue). (**E**). Comparative qRT-PCR expression analysis of DNA polymerase lambda (Polλ) and DNA polymerase beta (Polβ) in brain samples of Polμ^−/−^ and wild-type mice, at several ages (11, 14 and 19 months). All results were referred to the expression level demonstrated in the wild-type animals. Samples were run in triplicated, and several mice (3–5) were used for each determination, that was normalized using the internal actin expression control. Data are expressed as the mean value ± SD. (***,** *P*<0.0001, Student’s t test).
(TIF)

Figure S7 **Comparative evaluation of DNA repair activity in brain extracts.** **(A).** Evaluation of gap filling activity in brain (wild-type vs. Pol**µ** ^−/−^) extracts, using the indicated labeled-template primer (upper part). After incubation (30′–1 h, 30°C) with the clarified extracts (10 µg), with the addition of the indicated concentration of ddCTP, products were recovered and resolved in 20% PAGE. As an additional variable, we tested different combinations of divalent activation cation. The different observed products are indicated. The original labeled primer could appear (+15) intact or degraded by endogenous nucleases, meanwhile extended primers appear at position (+16). **(B, C**). Evaluation of the “repair” activity on the above indicated (A) template primer in brain (wild-type vs. Polµ^−/−^) extracts, using dNTPs **(B**) or rNTPs (**C**). The graphics show a quantification of the generation of the full-length (+34) “repaired” product (also indicated by an asterisk in Figure 4E). The figure also shows the preference of immediate insertion (+16) in the template primer used (**A**) of purified hPolµ enzyme (25 nM), using dNTPs (**D**) or rNTPs (**E**), and identical reaction conditions to (**B, C**). That profile found is quite similar with the revealed in the wild-type brain extracts (**B, C**).
(TIF)

Figure S8 **Evaluation of mitochondrial activity in brain.** Crude extract or mitochondrial fractions, prepared as previously described (*Birch-Machin and Turnbull, 2001*) from wild-type or Polµ^−/−^ brains, were evaluated for their mitochondria number (**A**), defined as citrate synthetase activity (CS IU) per mg of protein, or the mtDNA content (**B**), expressed as the ratio mtDNA/nDNA. Relative extract activity for Complex I (CI), Complex II (CII) and Complex I+III (CI+CIII) was monitored (**C, D** and **E**, respectively) and expressed in relation with total protein. All estimations were carried essentially as previously described (*Acín-Pérez et al., 2004*). Finally mitochondrial supercomplexes were also studied in wild-type or Polµ^−/−^ brain extracts from old (18 m) mice (n = 3). (**F**). Wild-type and Polµ^−/−^ mitochondrial brain fractions, solubilized with digitonin (4 mg DIG/mg protein), were separated in blue native gel electrophoresis (BNGE) and analyzed by western blot for CIII (core 2) –upper panel-, for CIV (COXI 2) –middle panel- and FpSDH. All determinations were done as previously described (*Acín-Pérez et al., 2008*). Comparisons between groups were made using one-way ANOVA. Pair wise comparisons were made by post hoc Fisher PLSD test. Differences were considered statistically significant at *P<*0.05; *P<0,01; **, *P*<0.001; ***, *P*<0.0001; ns, non significant. Data analyses were performed using the statistical program StatView. In all experiments, error bars indicate standard deviations (Adept Scientific, Bethesda, MD, USA).
(TIF)

Figure S9 **qRT-PCR expression analysis of a panel of aging-associated functions in brain of aged Polµ^−/−^ mice. (A).** Differential transcript levels of selected aging-related (*Zahn et al., 2006*) functions, related to inflammation (*C1qa, C5a, C3aR, C5aR, Cox2*), mitochondrial activity (*Timm17a, Nfa10, Mrps12, Mrpl28, Atp5a*), and apoptosis or p53 targets (*Acin1, Egln3, Bid, P21, P16, Perp, Pten*) were assessed by real-time reverse transcription polymerase chain reaction from brain samples of 17–19-month-old mice; each determination was normalized using the internal actin expression control, and represented as the level of expression of the indicated gene in Polµ^−/−^ mice (gray and dashed bars) relative to the expression level in age-paired wild-type animals (black bar), taken as 1. Some genetic functions seem to be unaffected by the genetic ablation of *Polµ* (denoted by dark gray bars), others are reduced (clear gray), or appear up-regulated (dashed bars). The figure shows the median result of at least three independent experiments; for the different gene functions the number of determinations varied (n = 4–8); (*) The expression of *Egln3* (*Phd3*) presents important differences only at very late ages (19 months and more); even in this case the differences (augmentation in the wild-type animals) is apparent in only a small percentage of the animals analyzed; (**) The expression levels of Cox2 is very variable between individuals. **(B)**. qRT-PCR expression analysis of a selection of genes (*Sox2, Snca, Hoxa3, Zic3, Hes1* and *Bmp4*) with a clear role in development or function of the neural system in brain of wild-type animals; brain samples of treated (p) or non-treated (-) animals with an acute dose of paraquat (50 mg/kg) and recovered 7 hours later. Each determination was normalized using the internal actin expression control; for the different gene functions the number of determinations varied (n = 4–8).
(TIF)

Figure S10 **Brain expression profile alterations associated to oxidative stress.** Differential mRNA expression analysis between wild-type and Polµ^−/−^ (12–14 m) mice, treated (c) or non-treated (s) with an acute unique dose (50 mg/Kg) of paraquat, and samples taken 7 h later. (**A**). Venn diagram showing the different relations. (**B**) Principal components analysis (PDA) of the unfiltered normalized data. Triangles represent data from untreated samples and squares dots from PQ-treated samples. Blue correspond to wild-type samples and red to Polµ^−/−^ samples. (**C**) Schematic representation of the expression networked detected using the software Ingenuity (Ingenuity Systems Inc) mainly associating several Hox genes.
(TIFF)

Table S1 (DOCX)

Text S1 (DOCX)
