# Supplementary figures and images for: Increased Learning and Brain Long-Term Potentiation in Aged Mice Lacking DNA Polymerase μ
Source: PLoS One. 2013 Jan 3;8(1):e53243. doi: 10.1371/journal.pone.0053243 (PMC3536760; doi:10.1371/journal.pone.0053243)

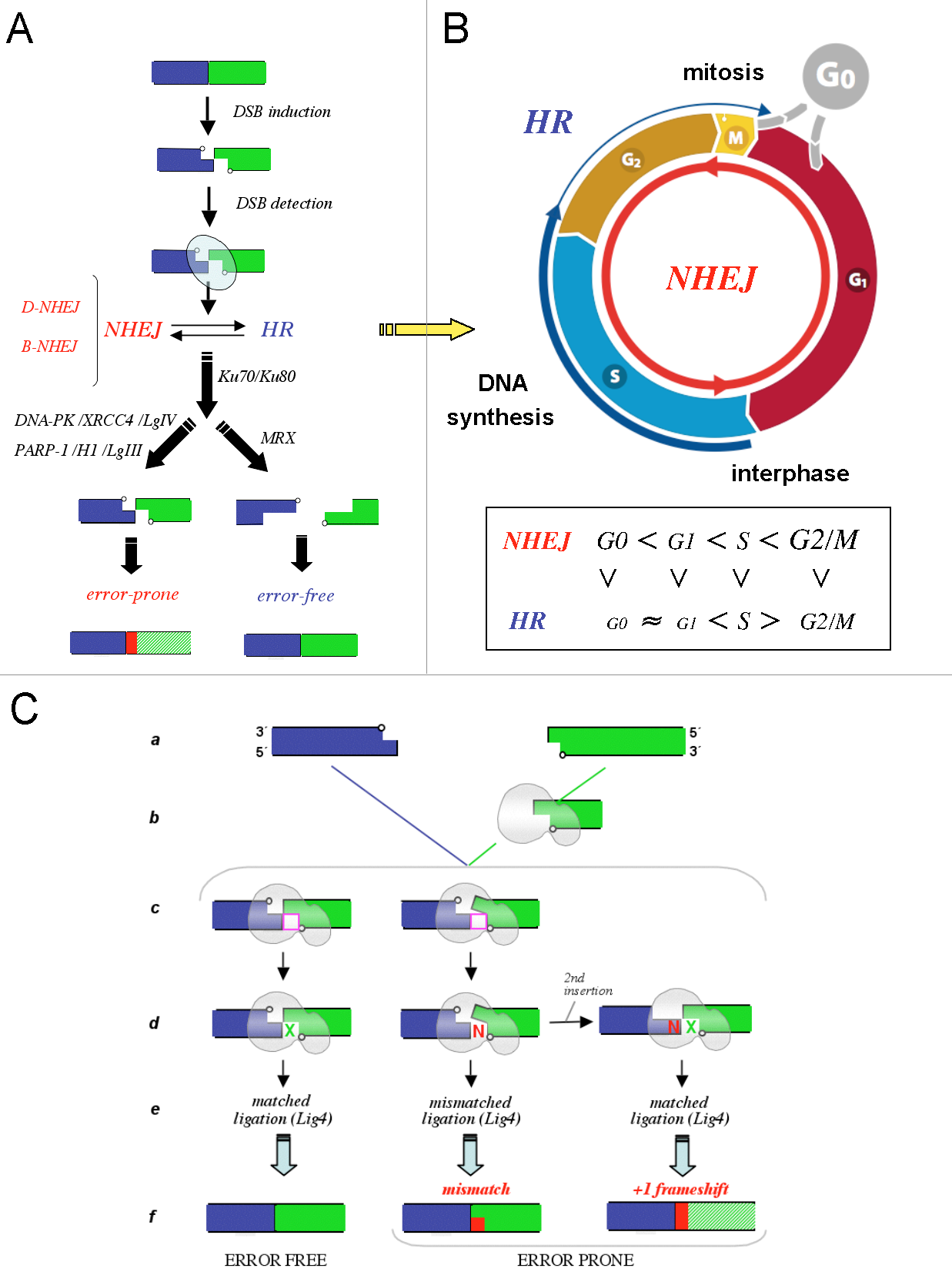

Supplement: Figure S1 — Mechanistic working model for in vivo Polµ function in NHEJ reactions. (A) NHEJ (error-prone) and HR (error-free) are the fundamental pathways for double-strand break (DSB) repair in mammals, and both compete for the same substrates. The contribution of either mechanism is dictated by the avidity of the heterodimer ku70/ku80 and the activity of the MRX complex for the DSB (Lee et al., 2008), and is strictly dependent on the cell cycle status; D-NHEJ, the classical pathway dependent on DNA-PK; B-NHEJ, a novel subpathway (called backup NHEJ mechanism), recently characterized, dependent on PARP-1, DNA Ligase III, and Histone 1 (Rosidi et al., 2008). (B) The HR pathway is preferentially active in the S and G2 phases of the cell cycle when a homologous sister chromosome or chromatid is available for direct base-pairing to effect error-free repair of a DNA DSB. Conversely, the NHEJ repair pathway can be used within any phase of the cell cycle and can be error-prone. The reported comparative strength of the two mechanisms (NHEJ vs. HR) along the different phases of the cell cycle has been schematized, following recent evidence (Kan’o et al., 2007; Mao et al., 2008; Natarajan et al., 2008). The letter size is intended to illustrate graphically the comparative contribution of the two mechanisms in each phase of the cell cycle. (C) A basic model for Polµ-dependent nucleotide insertions catalyzed during DSB repair. Processed NHEJ typically results in rearrangements. End-processing activities exist to deal with terminal damage, and occasionally, incompatible ends are generated as a collateral phenomenon. Polµ is unique in that it can process incompatible overhangs: at some end sequences, Polµ action can be template-directed (error-free), but in other cases wrong nucleotides are inserted, thus contributing to mutagenesis. (a) Schematic representation of a minimally processed DSB (1nt 3′-protruding ends), with non-complementary sequences. (b) Polµ structure (in gray) depicted as [file pone.0053243.s001.tif]

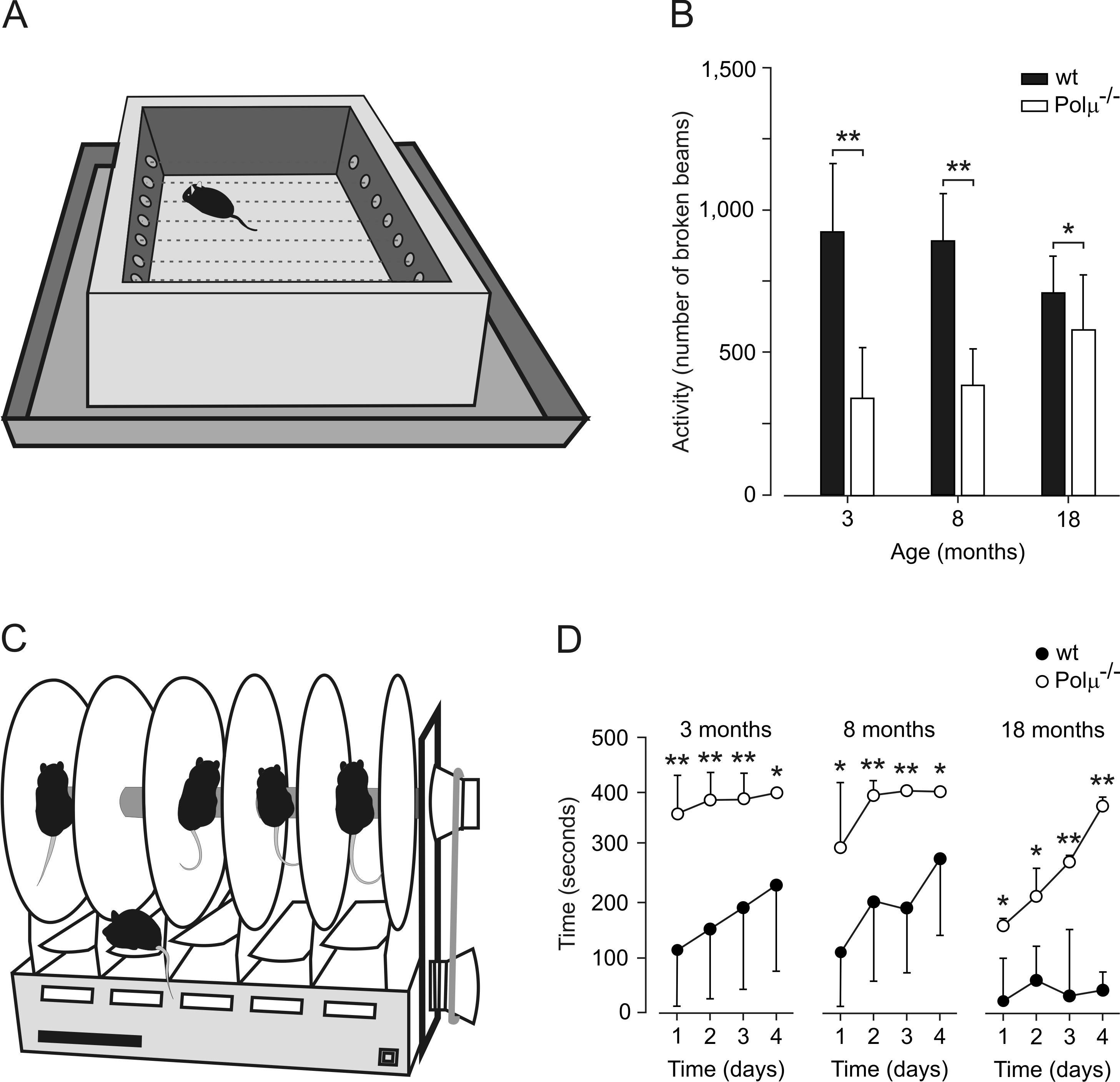

Supplement: Figure S2 — Polµ−/− mice display reduced exploratory activity and enhanced sensorimotor coordination during aging. (A–B) Motor activity (defined as number of beam interruptions per 10 min) of 3-, 8-, and 18-month-old wild-type (black bars) or Polµ −/− (white bars) mice. (C–D) Maximum time of permanency on the rota-rod (C) for 3-, 8-, and 18-month-old wild-type (closed circles) or Polµ −/− (open circles) mice analyzed on a rota-rod machine for 400 s per day over 5 days. (D) A total of n = 15 animals/group were used in these experiments. * P<0.05, ** P<0.01, two-way ANOVA. (TIF) [file pone.0053243.s002.tif]

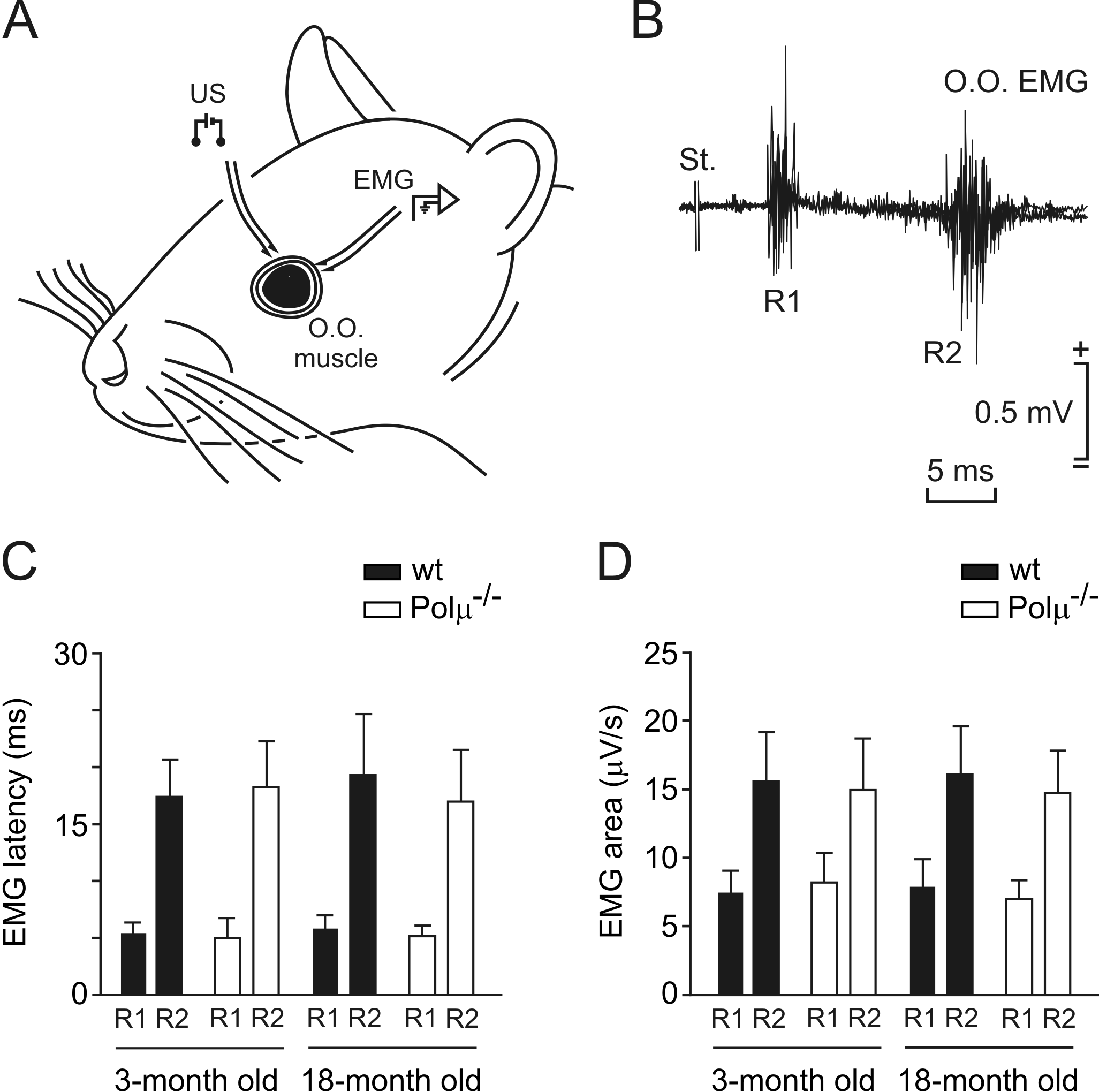

Supplement: Figure S3 — Characteristics of eyeblink responses evoked in young and aged Polµ−/− and wild-type mice. (A) A diagram indicating the location of stimulating (St.) electrodes implanted on the supraorbital nerve and electromyographic (EMG) recording electrodes implanted in the orbicularis oculi (O.O.) muscle. (B) Three superimposed records of the O.O. EMG response to the electrical stimulation of the ipsilateral supraorbital nerve collected from an 18-month-old Polµ −/− mouse. Note the two short- (R1) and long- (R2) latency components characterizing the blink reflex in mammals. EMG calibration as indicated. (C, D) Mean (± SEM; n = 20 measurements) values collected for EMG latency (C) and area of rectified EMG records (D) of both R1 and R2 components of electrically evoked blinks in 3- and 18-month-old wild-type (black bars) and Polµ −/− (white bars) mice. No significant difference (P≥0.425, two-way ANOVA) was observed between groups for any of the four parameters. (TIF) [file pone.0053243.s003.tif]

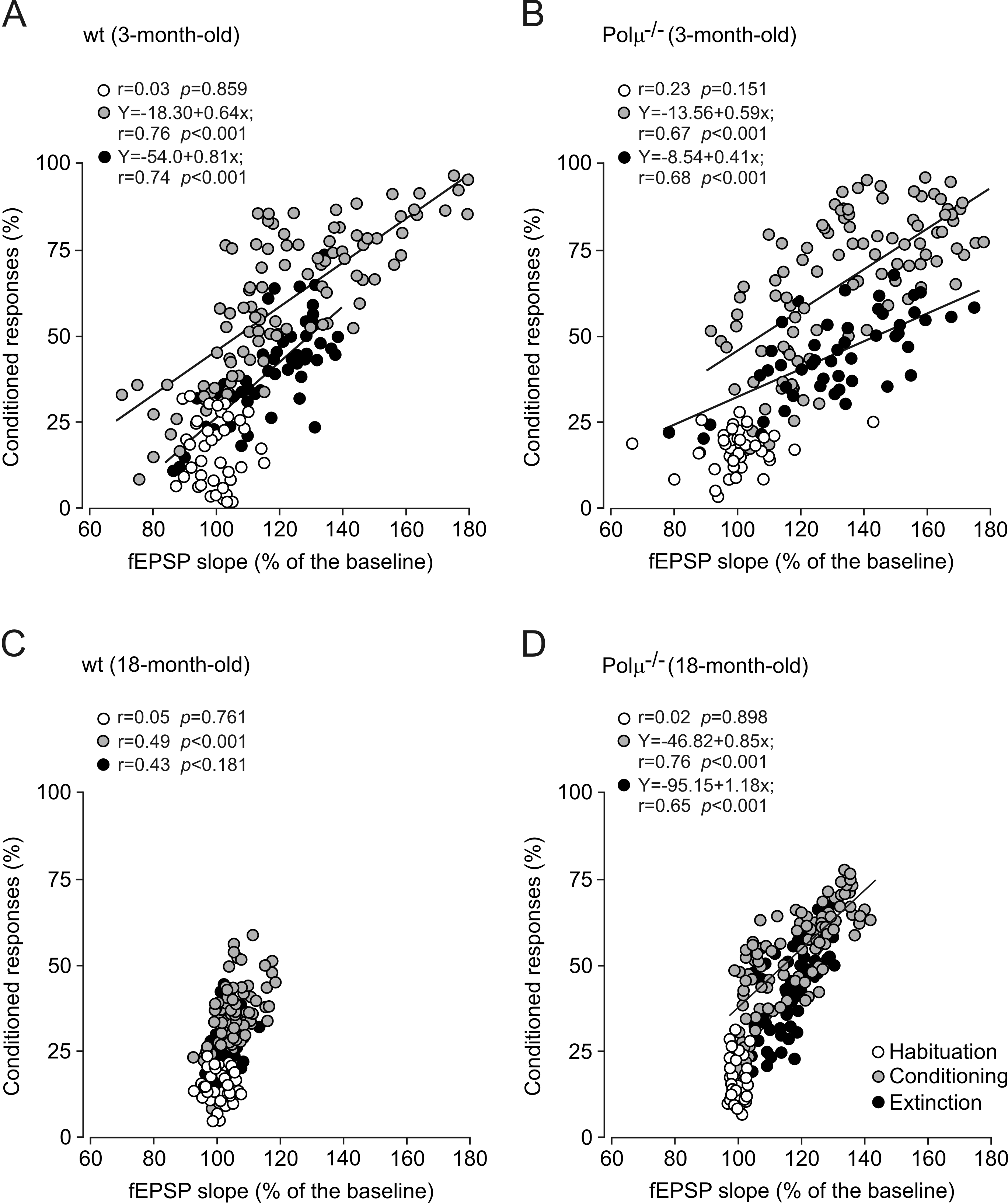

Supplement: Figure S4 — Quantitative analysis of the relationships between the percentage of CRs and fEPSP slopes for the different experimental groups across habituation, conditioning, and extinction sessions. Data collected from 3-month-old wild-type (A) and Polµ −/− (B) mice and from 18-month-old wild-type (C) and Polµ −/− (D) mice are illustrated. Each point represents the mean value collected from a single animal during the corresponding session. Equations corresponding to each of the three relationships included in each plot are indicated. Note that this linear regression analysis was non-significant for habituation sessions in all of the groups and for conditioning and extinction sessions as well in the 18-month-old control group. (TIF) [file pone.0053243.s004.tif]

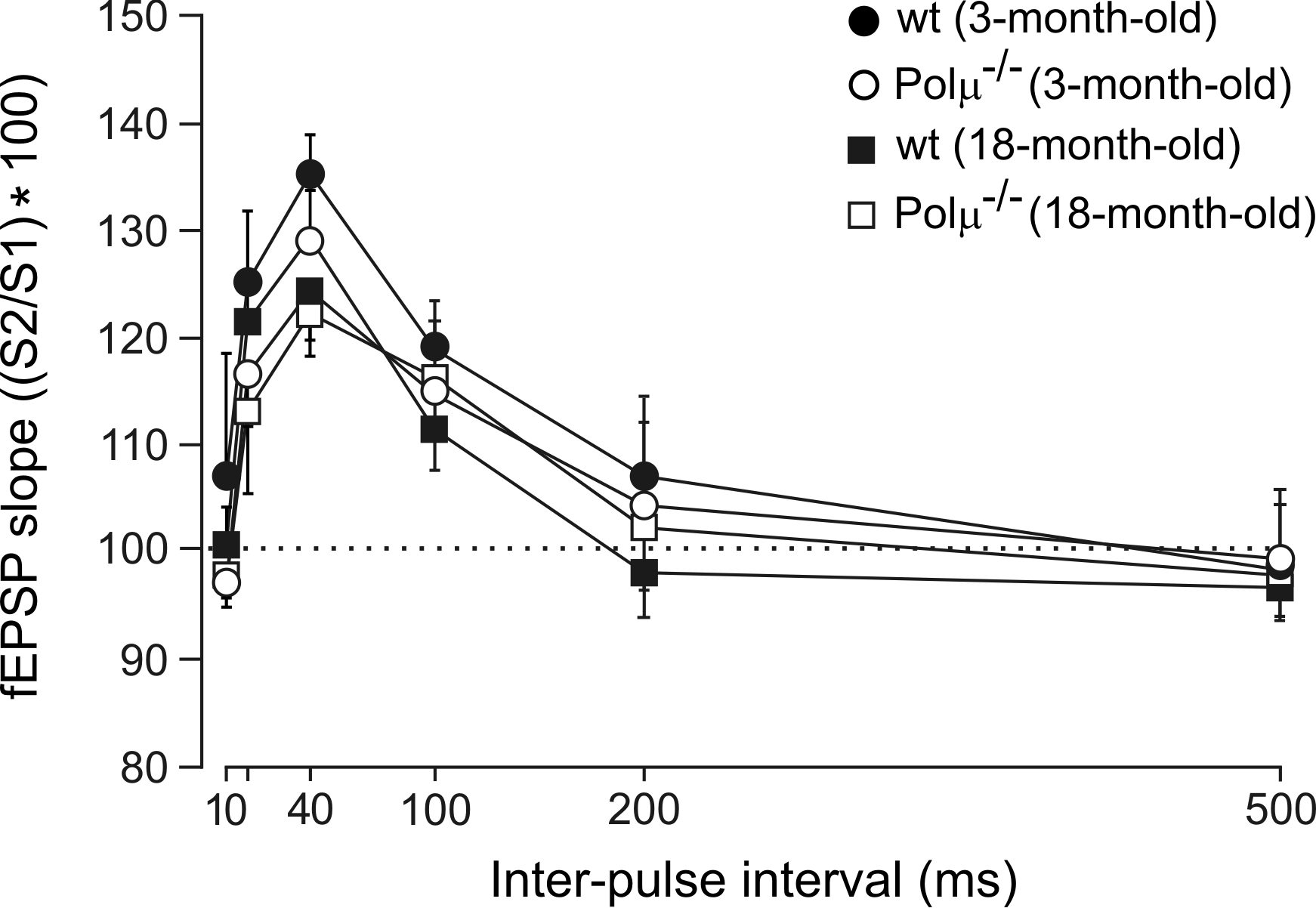

Supplement: Figure S5 — Paired-pulse facilitation of field excitatory postsynaptic potentials (fEPSP) recordings in the CA1 area following stimulation of the ipsilateral Schaffer collateral-commissural pathway. Data were collected from extracellular fEPSP paired traces collected from 18-month-old wild-type and Polµ −/− mice at different inter-pulse intervals. The data shown are mean ± SEM slopes of the second fEPSP expressed as a percentage of the first for the six (10, 20, 40, 100, 200, 500 ms) inter-stimulus intervals for the four experimental groups. (TIF) [file pone.0053243.s005.tif]

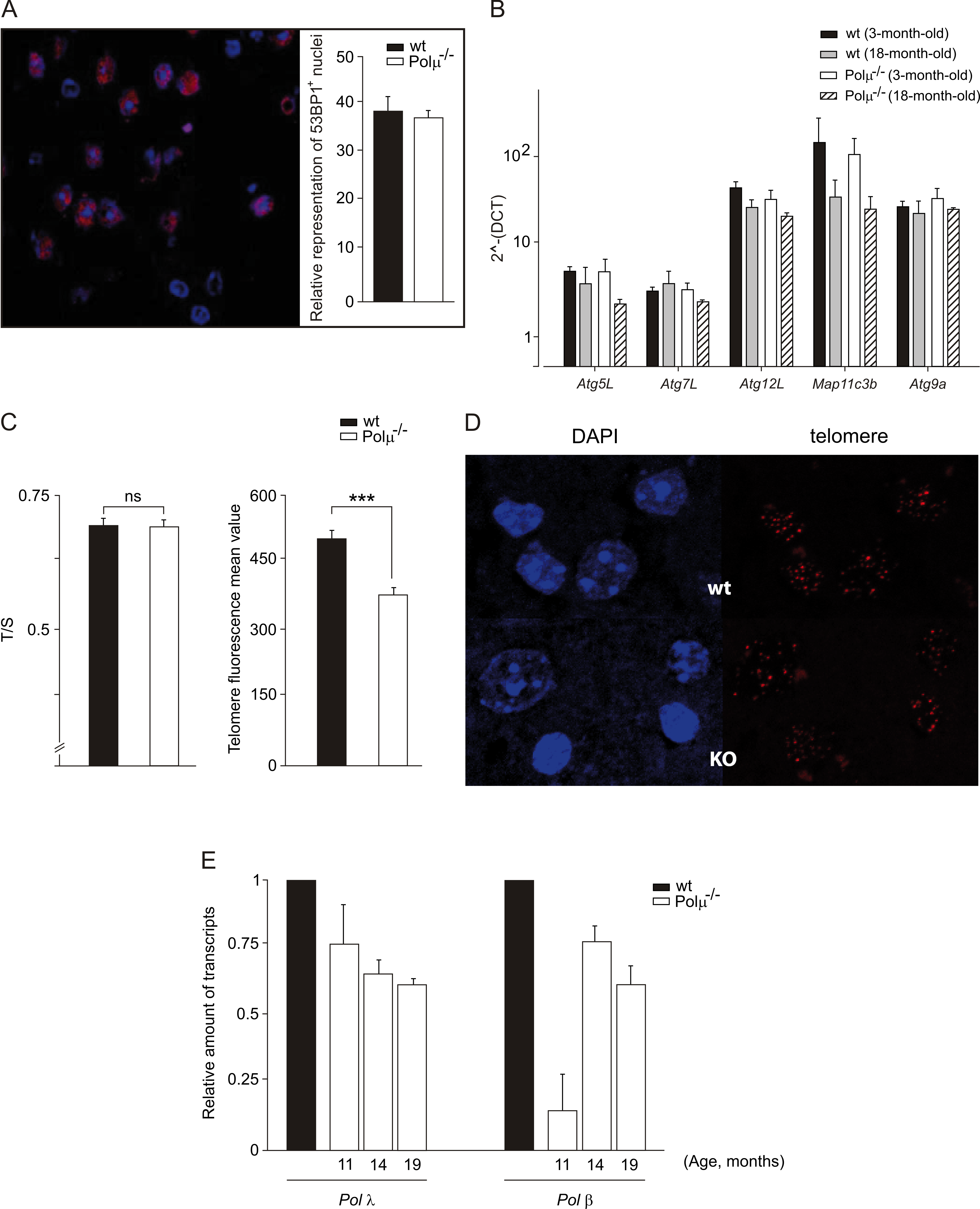

Supplement: Figure S6 — Molecular characterization of Polμ−/− mice associated with aging. (A) Paraffin brain sections from wild-type and Polµ −/− mice (18–20 months old) were processed and stained for 53BP1 (M). (B) qRT-PCR expression analysis of a selection of genes (Atg5l, Atg7l, Atg12l, Maplc3b, and Atg9a) critical for execution or regulation of autophagy. Black bars correspond to wild-type samples and gray bars to Polµ −/−. (C, D). Telomere-length evaluation in brain tissue. In C, genomic DNA from wild-type or Polµ −/− mice was subjected to telomere-specific PCR reaction using specific primers. The relative telomere length ratio (T/S) was calculated as indicated in Material and Methods section, and is defined as 2?-(delta Ct), where delta Ct = mean Ct telomere/mean Ct single copy internal gene. Quantitative FISH was carried out using a Cy3- labeled LL(CCCTAA)3 peptide nucleic acid (PNA) telomeric probe as previously described (Estrada et al., 2011). In D are illustrated representative pictures of wild-type and Polμ−/− samples stained for telomere length (red) and nuclei (DAPI, blue). (E). Comparative qRT-PCR expression analysis of DNA polymerase lambda (Polλ) and DNA polymerase beta (Polβ) in brain samples of Polμ−/− and wild-type mice, at several ages (11, 14 and 19 months). All results were referred to the expression level demonstrated in the wild-type animals. Samples were run in triplicated, and several mice (3–5) were used for each determination, that was normalized using the internal actin expression control. Data are expressed as the mean value ± SD. (***,** P<0.0001, Student’s t test). (TIF) [file pone.0053243.s006.tif]

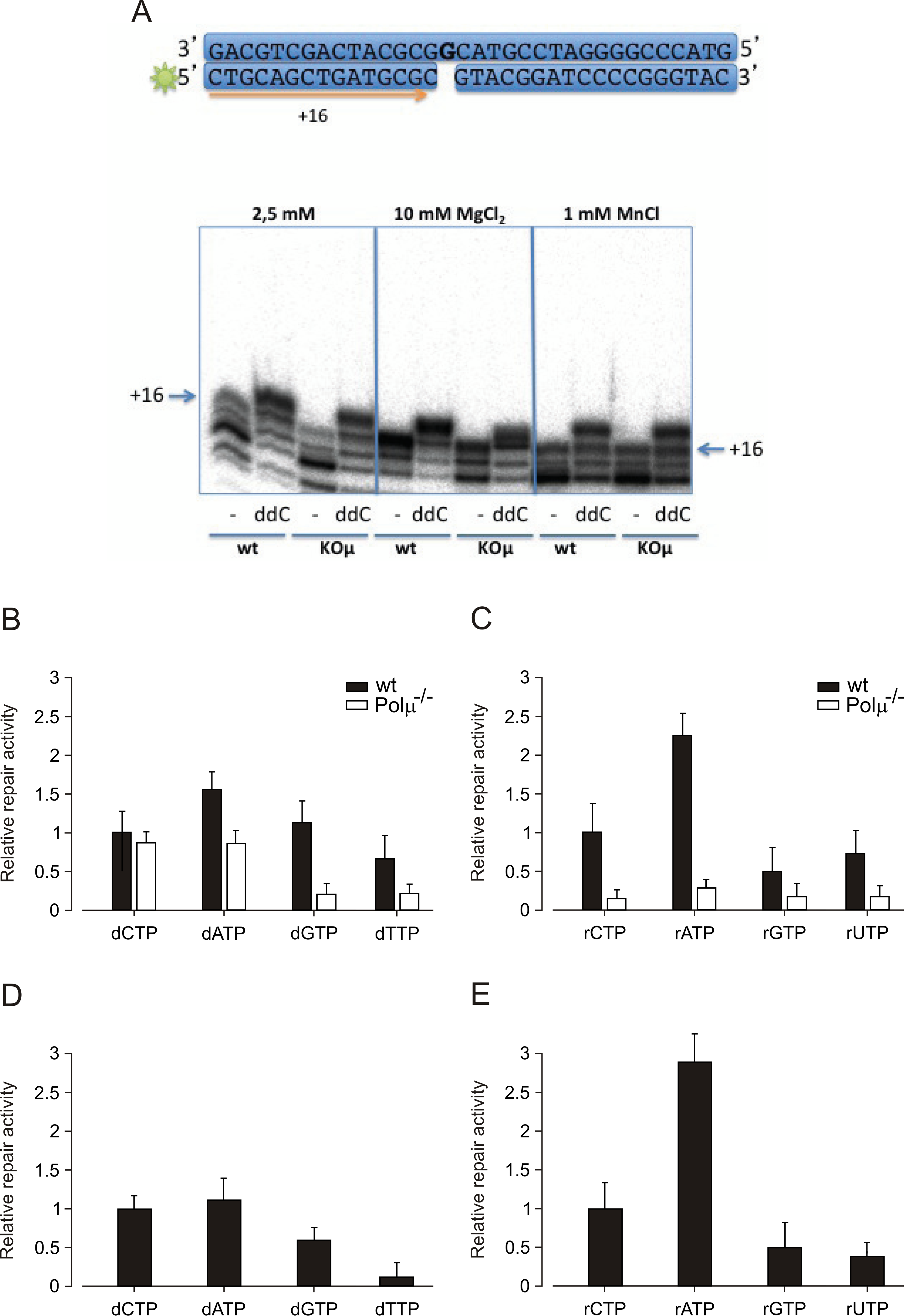

Supplement: Figure S7 — Comparative evaluation of DNA repair activity in brain extracts. (A). Evaluation of gap filling activity in brain (wild-type vs. Polµ −/−) extracts, using the indicated labeled-template primer (upper part). After incubation (30′–1 h, 30°C) with the clarified extracts (10 µg), with the addition of the indicated concentration of ddCTP, products were recovered and resolved in 20% PAGE. As an additional variable, we tested different combinations of divalent activation cation. The different observed products are indicated. The original labeled primer could appear (+15) intact or degraded by endogenous nucleases, meanwhile extended primers appear at position (+16). (B, C). Evaluation of the “repair” activity on the above indicated (A) template primer in brain (wild-type vs. Polµ−/−) extracts, using dNTPs (B) or rNTPs (C). The graphics show a quantification of the generation of the full-length (+34) “repaired” product (also indicated by an asterisk in Figure 4E). The figure also shows the preference of immediate insertion (+16) in the template primer used (A) of purified hPolµ enzyme (25 nM), using dNTPs (D) or rNTPs (E), and identical reaction conditions to (B, C). That profile found is quite similar with the revealed in the wild-type brain extracts (B, C). (TIF) [file pone.0053243.s007.tif]

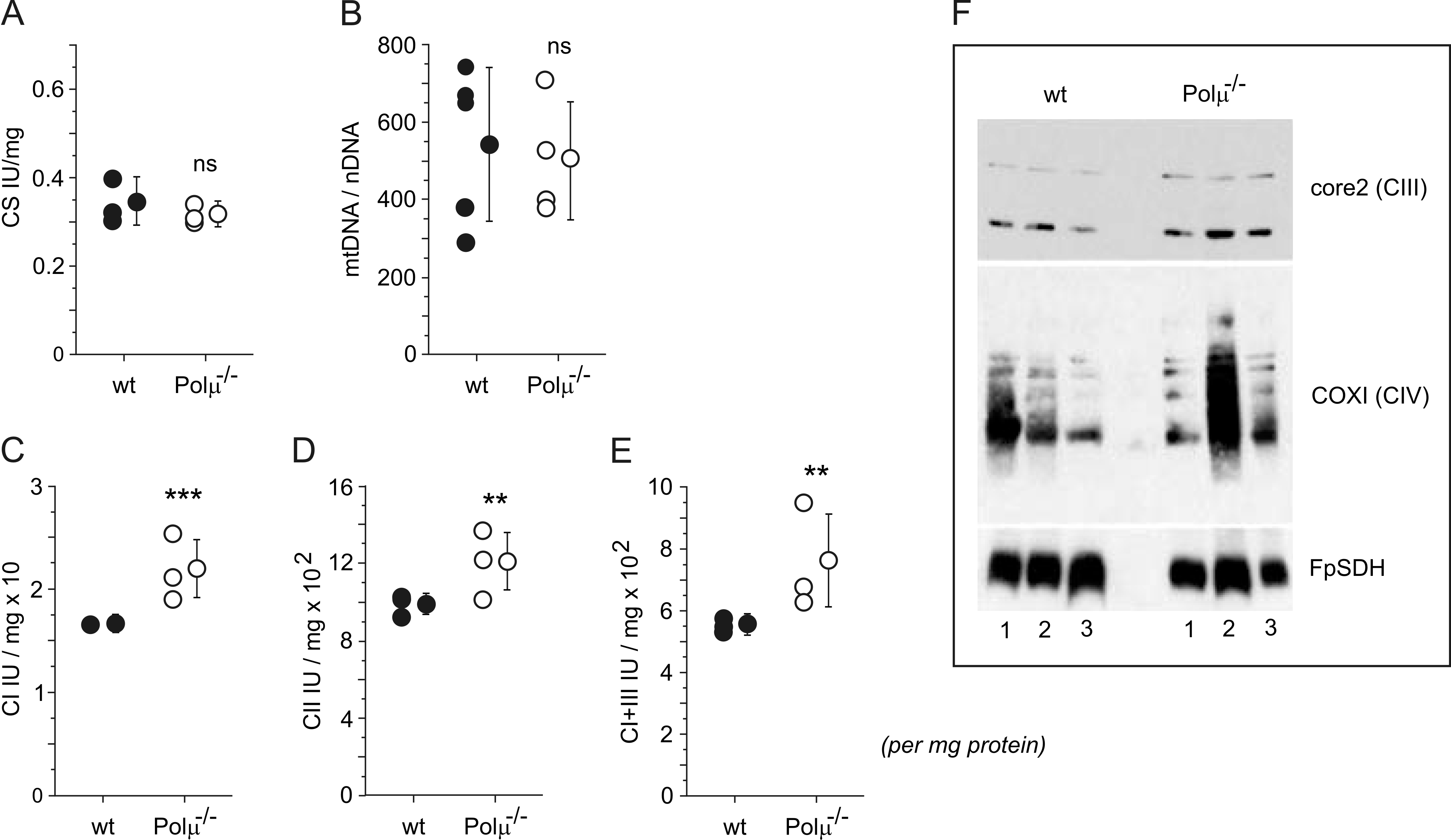

Supplement: Figure S8 — Evaluation of mitochondrial activity in brain. Crude extract or mitochondrial fractions, prepared as previously described (Birch-Machin and Turnbull, 2001) from wild-type or Polµ−/− brains, were evaluated for their mitochondria number (A), defined as citrate synthetase activity (CS IU) per mg of protein, or the mtDNA content (B), expressed as the ratio mtDNA/nDNA. Relative extract activity for Complex I (CI), Complex II (CII) and Complex I+III (CI+CIII) was monitored (C, D and E, respectively) and expressed in relation with total protein. All estimations were carried essentially as previously described (Acín-Pérez et al., 2004). Finally mitochondrial supercomplexes were also studied in wild-type or Polµ−/− brain extracts from old (18 m) mice (n = 3). (F). Wild-type and Polµ−/− mitochondrial brain fractions, solubilized with digitonin (4 mg DIG/mg protein), were separated in blue native gel electrophoresis (BNGE) and analyzed by western blot for CIII (core 2) –upper panel-, for CIV (COXI 2) –middle panel- and FpSDH. All determinations were done as previously described (Acín-Pérez et al., 2008). Comparisons between groups were made using one-way ANOVA. Pair wise comparisons were made by post hoc Fisher PLSD test. Differences were considered statistically significant at P<0.05; *P<0,01; **, P<0.001; ***, P<0.0001; ns, non significant. Data analyses were performed using the statistical program StatView. In all experiments, error bars indicate standard deviations (Adept Scientific, Bethesda, MD, USA). (TIF) [file pone.0053243.s008.tif]

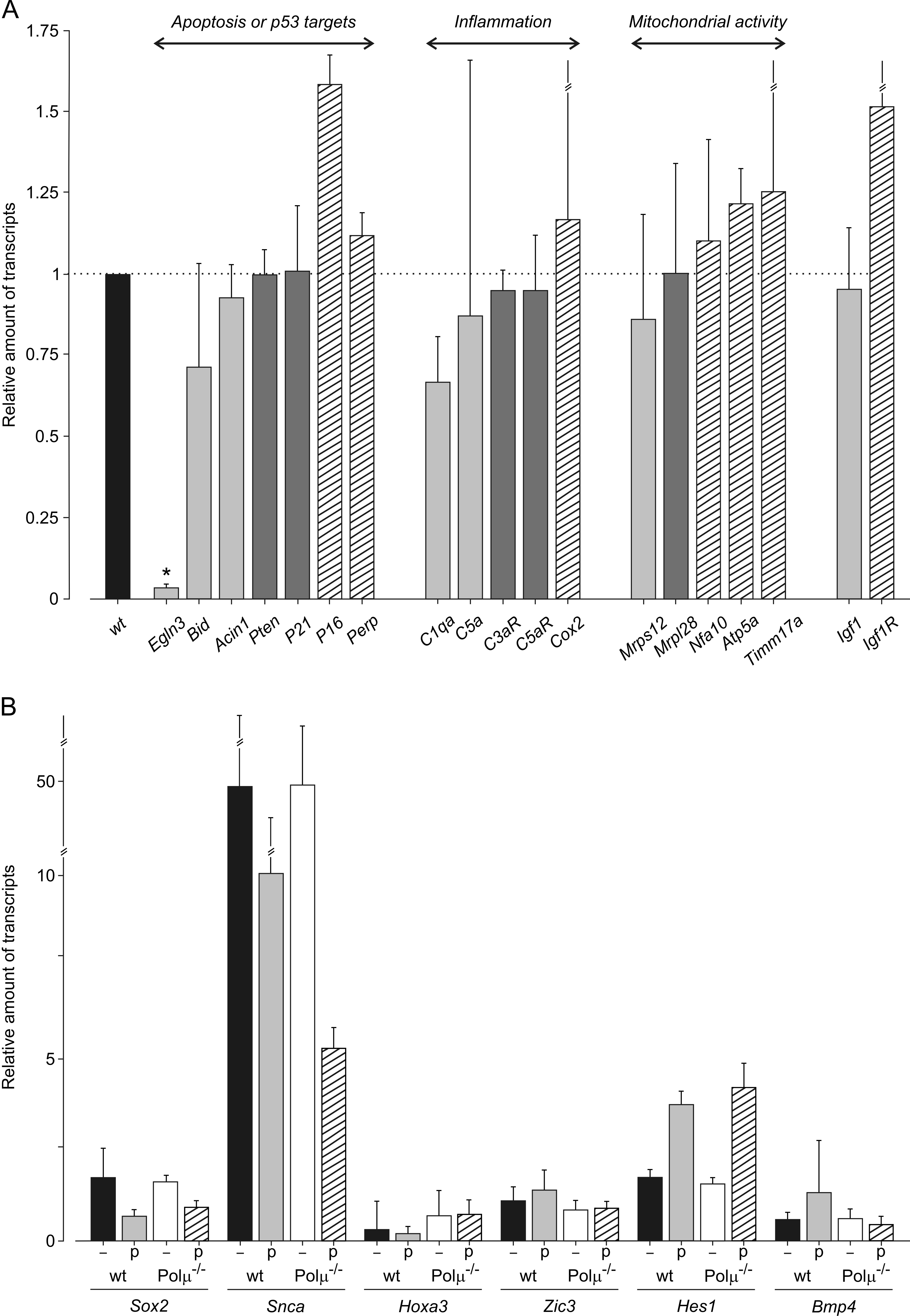

Supplement: Figure S9 — qRT-PCR expression analysis of a panel of aging-associated functions in brain of aged Polµ−/− mice. (A). Differential transcript levels of selected aging-related (Zahn et al., 2006) functions, related to inflammation (C1qa, C5a, C3aR, C5aR, Cox2), mitochondrial activity (Timm17a, Nfa10, Mrps12, Mrpl28, Atp5a), and apoptosis or p53 targets (Acin1, Egln3, Bid, P21, P16, Perp, Pten) were assessed by real-time reverse transcription polymerase chain reaction from brain samples of 17–19-month-old mice; each determination was normalized using the internal actin expression control, and represented as the level of expression of the indicated gene in Polµ−/− mice (gray and dashed bars) relative to the expression level in age-paired wild-type animals (black bar), taken as 1. Some genetic functions seem to be unaffected by the genetic ablation of Polµ (denoted by dark gray bars), others are reduced (clear gray), or appear up-regulated (dashed bars). The figure shows the median result of at least three independent experiments; for the different gene functions the number of determinations varied (n = 4–8); (*) The expression of Egln3 (Phd3) presents important differences only at very late ages (19 months and more); even in this case the differences (augmentation in the wild-type animals) is apparent in only a small percentage of the animals analyzed; (**) The expression levels of Cox2 is very variable between individuals. (B). qRT-PCR expression analysis of a selection of genes (Sox2, Snca, Hoxa3, Zic3, Hes1 and Bmp4) with a clear role in development or function of the neural system in brain of wild-type animals; brain samples of treated (p) or non-treated (-) animals with an acute dose of paraquat (50 mg/kg) and recovered 7 hours later. Each determination was normalized using the internal actin expression control; for the different gene functions the number of determinations varied (n = 4–8). (TIF) [file pone.0053243.s009.tif]

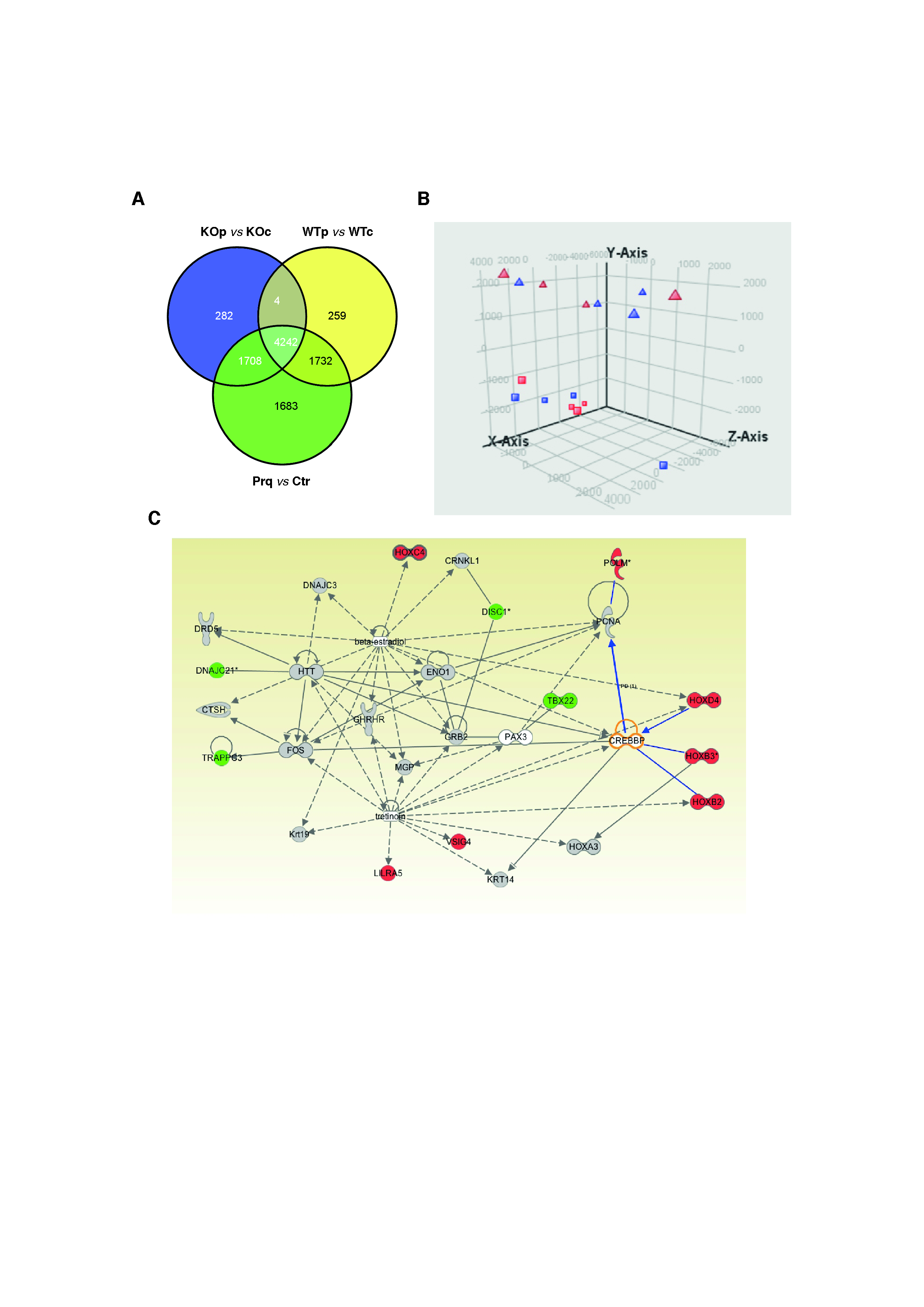

Supplement: Figure S10 — Brain expression profile alterations associated to oxidative stress. Differential mRNA expression analysis between wild-type and Polµ−/− (12–14 m) mice, treated (c) or non-treated (s) with an acute unique dose (50 mg/Kg) of paraquat, and samples taken 7 h later. (A). Venn diagram showing the different relations. (B) Principal components analysis (PDA) of the unfiltered normalized data. Triangles represent data from untreated samples and squares dots from PQ-treated samples. Blue correspond to wild-type samples and red to Polµ−/− samples. (C) Schematic representation of the expression networked detected using the software Ingenuity (Ingenuity Systems Inc) mainly associating several Hox genes. (TIFF) [file pone.0053243.s010.tiff]
