# Supplementary material for: Increased Learning and Brain Long-Term Potentiation in Aged Mice Lacking DNA Polymerase μ
Source: PLoS One. 2013 Jan 3;8(1):e53243. doi: 10.1371/journal.pone.0053243 (PMC3536760; doi:10.1371/journal.pone.0053243)
Supplement: Table S1 — (DOCX) [file pone.0053243.s011.docx]

**Table S1. Primers used for RT-PCR amplification**

| ***Atg5L*** | ATG5L Fw  ATG5L Rv | ACAGCTTCTGGATGAAAGGC  TGGGACTGCAGAATGACAGA |
| --- | --- | --- |
| ***Atg7L*** | ATG7L Fw  ATG7L Rv | GCCAGGTACTCCTGAGCTGT  GGTCTTACCCTGCTCCATCA |
| ***Atg9a*** | ATG9a Fw  ATG9a Rv | AAGGCAACCACAAAGAGGAA  GGCACCACATTGAAAACCTT |
| ***Atg12L*** | ATG12L Fw  ATG12L Rv | CCACAGCCCATTTCTTTGTT  GTCCTCGGCTGCAGTTTC |
| ***Bmp4*** | Bmp4Fw  Bmp4 Rv | TGGACTGTTATTATATGCCTTGTTTT  CTCCTAGCAGGACTTGGCAT |
| ***Erdr1*** | Erdr1 Fw  Erdr1 Rv | TTCACGCCCACAGAGAAACT  TTTAGCCGCAGCTATGGTTT |
| ***GH*** | Gh Fw  Gh Rv | CTTGAGGATCTGCCCAACAC  CCTCGGACCGTGTCTATGAG |
| ***Hes1*** | Hes1 Fw  Hes1 Rv | GCTTCCAAGTGGTGCCGGCT  TAGACCGGGATGACCGGGCC |
| ***Hoxa3*** | Hoxa3 Fw  Hoxa3 Rv | TCTTAACATGGAGG GAGCCA  TCTGAAGGCTACGTGTGCTG |
| ***Hoxb3*** | Hoxb3Fw  Hoxb3 Rv | ATCTGTTTGGTGAGGGTGGA  CCGCACCTACCAGTACCACT |
| ***Igfbp1*** | Igfbp1 Fwb  Igfbp1 Rvb | CTGCCAAACTGCAACAAGAATG  GGTCCCCTCTAGTCTCCAGA |
| ***Igfbp3*** | Igfbp3 Fwb  Igfbp3 Rvb | GTTGGGAGGGGAGGTAGGT  GAGCAGTACCCGCTGAGG |
| ***Map11c3b*** | Map11c3b Fw  Map11c3b Rv | GCTGCTTCTCCCCCTTGTAT  CCGAGAAGACCTTCAAGCAG |
| ***NipSNap1*** | Nipsnap1 Fw  Nipsnap1 Rv | GGAACCAGCTTCCTTCACTG  CTATTCACGAAGCCGAGACC |
| ***Pol beta*** | PolβFw  PolβRv | CCCATCCCAGCTTCACTTC  CCCGGTATTTCCACTGGAT |
| ***Pol lambda*** | PolλFw  PolλRv | CGAGCCAGAAGGCAACTAAC  TCTCCAGGATCTCCATGACC |
| ***Prl1*** | Prl1 Fw  Prl1 Rv | CTGCACCAAACTGAGGAT  CAATGACTGCCCCACTTC |
| ***Snca*** | SncaFw  SncaRv | CAGGCATGTCTTCCAGGATT  GGGAATATAGCTGCTGCCAC |
| ***Sox2*** | Sox2 Fw  Sox2 Rv | TGCTGCCTCTTTAAGACTAGGGCTG  CGCGGTCCGGGCTGTTCTTC |
| ***Zic3*** | Zic Fw  Zic Rv | AACCGTCTGTCACAGCCTTC  AAGATTTTTGCCCGCTCTG |
| ***Zmat5*** | Zmat5 Fw  Zmat5 Rv | GGAAGGAGCGGTCACAGTAG  CGAGGACTCAAGAGGAGGAG |
