# Supplementary material for: Increased Learning and Brain Long-Term Potentiation in Aged Mice Lacking DNA Polymerase μ
Source: PLoS One. 2013 Jan 3;8(1):e53243. doi: 10.1371/journal.pone.0053243 (PMC3536760; doi:10.1371/journal.pone.0053243)
Supplement: Text S1 — (DOCX) [file pone.0053243.s012.docx]

**Text S1 for**

**DNA polymerase μ deficiency retards brain aging and facilitates cognitive processes in aged mice**

**Daniel Lucas,^1,2^ José M. Delgado-García,^3^ Beatriz Escudero,^4,5^  Carmen Albo,^4,5^ Ana Aza,^6^ Rebeca Acín-Pérez,**^4,5^ **Yaima Torres,^4,5^ Paz Moreno,**^4^ **José Antonio Enríquez,**^4,5^ **Enrique Samper,^4,5^ Luis Blanco,^6^ Alfonso Fairén,**^7^ **Antonio Bernad, ^4,5,8,#,^* Agnès Gruart, ^3,#,^***

^1^Department of Immunology and Oncology, Centro Nacional de Biotecnología/CSIC, Madrid 28049, Spain. ^2^Current address; Albert Einstein College of Medicine, Bronx, New York, NY, USA. ^3^División de Neurociencias, Universidad Pablo de Olavide, 41013-Sevilla, Spain. ^4^Department of Regenerative Cardiology, Centro Nacional de Investigaciones Cardiovasculares, 28029-Madrid, Spain. ^5^Development and Cardiac Repair Department, Centro Nacional de Investigaciones Cardiovasculares, 28029-Madrid, Spain. ^6^Centro de Biología Molecular Severo Ochoa, Campus de Cantoblanco, 28049-Madrid, Spain. ^7^Instituto de Neurociencias, Consejo Superior de Investigaciones Científicas - Universidad Miguel Hernández, 03550-San Juan de Alicante, Spain. ^8^Translational Research Platform, Centro Nacional de Investigaciones Cardiovasculares, 28029-Madrid, Spain. **^#^**These authors share senior authorship.

*To whom correspondence should be addressed. E-mail: [agrumas@upo.es](mailto:agrumas@upo.es)

[abernad@cnic.es](mailto:abernad@cnic.es)

**This PDF file includes**

A. Supplementary information

A.1. Antecedents

A.2. Supplementary results

B. References

**A. Supplementary information**

**A.1. Antecedents**

Double-strand breaks (DSB) in DNA arise due to a combination of endogenous and exogenous factors during cell life (*Lieber et al., 2004*). DSB are one of the most deleterious forms of DNA damage, and several mechanisms have evolved to repair them. In eukaryotes, the two main DSB repair pathways are homologous recombination (HR) and non-homologous end-joining (NHEJ) (*Lieber et al., 2004;* see scheme in Figure S1A). HR uses information from the homologous allele to direct repair of the damaged strand. In NHEJ, an intact allele is not available, and the two ends of the break are joined directly or reconnected in a process guided by microhomology (short patches of homology on both ends of the break); as a consequence, NHEJ-repaired DSB are prone to mutations/deletions (for a recent review see *Sonoda et al., 2006*). Whereas HR is prevalent in yeast, NHEJ is the most frequent DSB repair mechanism in higher eukaryotes, and is used preferentially in G1 and early S phases of the cell cycle (*Sonoda et al., 2006;* see scheme in Figure S1A, B).

The NHEJ process has been well characterized in recent years. Most of the essential factors have been discovered, and include the Ku70/80 heterodimer, the DNA protein kinase catalytic subunit (DNA-PKcs), and the XRCC4-ligaseIV complex (*Sonoda et al., 2006*; *Ma et al., 2004*). Two additional factors were recently reported ― the Artemis nuclease (*Moshous et al., 2001*) and the XLF/Cernunnos protein (*Ahnesorg et al. 2006*; *Buck et al. 2006*). All these factors catalyze end-joining reactions *in vitro*, and mice deficient in any one of them show alterations in the DSB repair response (radiosensitivity) and in V(D)J recombination, a specialized NHEJ reaction (*Lieber et al., 2004*). Despite their critical role in DSB repair, only XRCC4 (*Gao et al. 1998a,b*) or ligase IV deficiencies are embryonic lethal (*Barnes et al. 1998*; *Frank et al. 1998*), due to extended neuronal apoptosis; inactivation of p53 rescues embryonic lethality in LigIV^-/-^ mice, but not lymphopoiesis impairment or radiosensitivity (*Frank et al., 2000*). DNA‑PKcs-deficient mice have impaired V(D)J recombination and are radiosensitive (*Gao et al., 1998a,b*; *Taccioli et al., 1998*; *Kurimasa et al., 1999*). Ku70^-/-^ mice show growth retardation, impaired V(D)J recombination, increased incidence of thymic lymphomas, and premature senescence in mouse embryonic fibroblasts (MEF) (*Gu et al., 1997*; *Ouyang et al., 1997*). Ku80 deficiency results in increased MEF doubling time, premature senescence, genetic instability, V(D)J recombination impairment, and growth retardation (*Nussenzweig et al., 1996;* *Zhu et al., 1996*). The common phenotypes in NHEJ-deficient mice are thus altered V(D)J recombination, radiosensitivity, and genetic instability.

After strand alignment in the NHEJ reaction, some breaks may present gaps that must be filled by DNA polymerase activities. Although the requirement for a DNA polymerase during end-joining was shown a few years ago (*Lieber et al., 2004*), the identification of this factor(s) remained elusive in higher eukaryotes, probably due to a certain redundancy among polymerases. There are currently two clear candidates for NHEJ polymerases ― Polμ and Polλ― both members of the X family of DNA polymerases (*Moon et al., 2007a,b*). Polλ is very similar to Pol4, the *Saccharomyces cerevisiae* NHEJ polymerase, whereas Polμ is the closest relative to the terminal transferase enzyme (TdT), which acts during the V(D)J NHEJ reaction. Polμ and Polλ interact with Ku, XRCC4, and LigIV factors (*Mahajan et al., 2002; Ma et al., 2004*), and both enzymes participate in Ku-dependent *in vitro* end-joining reactions (*Ma et al., 2004*). Polμ and Polλ have been shown to play different roles in V(D)J recombination (*Bertocci et al., 2003*; *Nick McElhinny et al., 2005*; *Bertocci et al., 2006*), suggesting their implication in different and specialized end-joining reactions. Recently, in a cell model with an integrated NHEJ substrate, overexpression of catalytically inactive Polλ resulted in altered DSB repair, suggesting that Polλ can participate in NHEJ reactions (*Capp et al., 2006*).

Polμ is an error-prone DNA-directed DNA polymerase (*Aoufouchi et al., 2000; Domínguez et al., 2000;* *Ruiz et al., 2001, 2004; Juárez et al., 2006*) and is expressed in many tissues (liver, kidney, lung, brain, and testis, although it is especially abundant in lymphohematopoietic organs (*Domínguez et al., 2000, Lucas et al., 2005*). In addition, Polμ interacts with different components of the NHEJ repair pathway (Ku 70/80) and is up-regulated after double-strand break (DSB) induced by γ-irradiation, a known clastogen (*Mahajan et al., 2002*). Staining with anti-phosphorylated γ-H2AX, a modified histone found at DSB sites, revealed Polμ recruitment to DSB (*Mahajan et al., 2002*). *In vitro,* Polμ has been shown to participate in specific NHEJ reactions that require a DNA polymerase activity (*Mahajan et al., 2002, Nick McElhinny et al., 2005, Capp et al., 2007*). Furthermore, Polμ demonstrated unusual catalytic features (*Nick McElhinny and Ramsden, 2003; Ruiz et al., 2001, 2004*) that led to the proposal that, in some scenarios, could be a DNA-directed DNA/RNA polymerase (*Nick McElhinny and Ramsden, 2003)* because of its low discrimination against rNTPs at concentration closed to the supposed in vivo ones. These characteristic, taken together with the fact that Lig IV is capable to negotiate DSB ends with rNTPs incorporated, prompted to speculate that such an activity would have important consequences in the context of cellular double-strand break repair (*Nick McElhinny and Ramsden, 2003; Ruiz et al., 2001, 2004*). These and more recent results (*Juárez et al., 2006*) and the resolution of its crystal structure (*Moon et al., 2007a,b*) has allowed the establishment of a general working mechanistic hypothesis (Figure S1C).

Analysis of mice lacking DNA polymerase μ showed a specialized function for this enzyme during the V(D)J recombination process (*Bertocci et al., 2003*). Specifically Polμ was required for correct recombination of the immunoglobulin light chain κ, and its deficiency results in shorter, non-productive V_κ_-J_κ_ junctions, and hence in lymphocyte cell death at the ProB-to-PreB transition stage (*Bertocci et al., 2003*). Because the V(D)J recombination reaction is essentially a modified end-joining reaction (reviewed by *Soulas-Sprauel et al., 2007*), Polμ participation in this process demonstrated it is a requirement for (at least some) *in vivo* NHEJ reactions.

Polμ expression patterns (*Domínguez et al., 2000; Lucas et al., 2005*) suggested that Polμ might participate in NHEJ in other tissues, both during fetal development (*Gozalbo-López, 2009*) and in the adult organism, especially in non-B-cell populations in the hematopoietic system (*Lucas et al., 2009*), but also in the neural system. An important role for NHEJ during CNS development (*Karanjawala et al., 2003; Orii et al., 2006*), homeostasis (*Karanjawala et al., 2002; Neema et al., 2005*), and in various neural pathologies (*Holcomb et al., 2006; Liu et al., 2008)* has been already demonstrated, in both rodent and human cells (reviewed in *Lee and McKinnon, 2007*). Furthermore, several observations have indicated that NHEJ activity seems to decay during aging (*Ren and Peña de Ortiz, 2002; Vyjayanti and Rao, 2006; Rao et al., 2007; Sharma, 2007*). Finally, an NHEJ involvement and recombination activities have also been proposed to be somehow involved in learning and long-term memory generation (*Wang et al., 2003*). The latter is the center of a long-lasting debate since the seminal observation of the expression of recombination activating gene-1 (RAG-1) in brain (*Chun et al., 1991*). RAG-1 gene expression is restricted to neurons in the hippocampal formation and related limbic regions that are involved in spatial learning and memory as well as other parameters of neurobehavioral performance. Since then, sporadic confirmation of the original observation (*Sun et al., 2007*), and an increasing number of reports of rearranged and aneuploid chromosomes in living *in vivo* brain cells, strongly suggest an unexpected link between developmental chromosomal instability and brain genome diversity and evolution (reviewed by *Gericke, 2008*). Limited data provided support for the working hypothesis, but neurobehavioral alterations have been clearly established in RAG-1 knockout mice (*Cushman et al., 2003*). However, because in this mouse model both the neural and immune systems are affected, a mixed-crossed phenotype might be taking place (e.g., the loss of cytokines with neuromodulatory activities), so that definitive conclusions cannot be established. Therefore, the debate about the potential involvement of a novel, unknown mechanism, based on the controlled management of genomic instability/modification, in selected areas of the mammalian brain, coupled to maturation, learning, or other homeostatic/physiological processes, is still alive.

The results presented in this manuscript demonstrate that genetic elimination of Polμ, a novel accessory partner in the non-homologous end-joining (NHEJ) repair pathway for double-strand breaks (DSB), generates an unexpected phenotype of retarded brain aging. This phenotype concurs with activity-dependent changes taking place at the hippocampal CA3–CA1 synapse in conscious animals during eyeblink conditioning; aged Polμ^-/-^ mice acquired this associative test significantly better than wild-type littermate controls. Moreover, we were able to evoke LTP in behaving aged Polμ^-/-^ mice, but not in controls of the same age. Thus, these data sustain a role for the NHEJ machinery in the maintaining of brain functionally throughout the whole life. Remarkably, this is the first example in which the genetic ablation of a DNA repair function provokes a substantial better maintenance of cognitive/learning abilities, and of the concomitant activity-dependent synaptic plasticity at hippocampal circuits, in aged mice, together with lower signs of brain aging. Therefore, these results open a new avenue for studying the contribution or collaboration of error-prone repair mechanisms (such as those plausibly participated in by Polμ^-/-^) in mammalian physiological aging mechanisms.

**A.2. Results**

**Reflexively evoked eyelid responses are similar in young and aged wild-type and transgenic mice**

To rule out the possibility that the deficits in the acquisition of eyelid CRs could be related to age-dependent alterations of eyeblink circuits, we performed a series of preliminary experiments aimed at determining the functional characteristics of the corneal reflex evoked in young and aged Polμ^-/-^ and wild-type mice. Electrical stimulation (2 × threshold) of the supraorbital nerve evoked an early EMG activation of the orbicularis oculi muscle with a latency of 4-5 ms, followed by a second EMG activation, with latency from the stimulus of 15-20 ms (Figure S4A, B). These two successive muscle activations corresponded to the R1 and R2 components already described in humans (*Kugelberg, 1952*) and other species of mammals, including mice (*Domínguez del Toro et al., 2004; Porras-García et al., 2005; Gruart et al., 2006*). A quantitative analysis of the latency of R1 and R2 components of reflexively evoked blinks in young and old animals (n=5 animals per group; n=20 measurements) reveals no significant differences between wild-type and Polμ^-/-^ mice (*F*_(3,57)_ = 0.721; *P* = 0.544; Figure S4C). Similarly, no significant differences (*F*_(3,57)_ = 0.946; *P* = 0.425; Figure S4D) were found between wild-type and Polμ^-/-^ mice of the same age for the area of the rectified EMG records including the two R1 and R2 components of the blink reflex. Thus, blink reflex properties, with respect to latency and area of the rectified EMG recordings — which is linearly related to blink amplitude, see (*Evinger et al., 1991*) — seem to be unaffected by either age or the genetic manipulations made in Polμ^-/-^ mice.

**Activity-dependent synaptic plasticity at the CA3-CA1 synapse is linearly related to the increase in associative learning in young wild-type and in young and old Polμ^-/-^ mice, but not in old wild-type animals**

It has already been shown in wild-type C57Bl/6 mice that activity-dependent synaptic changes taking place within the hippocampal circuit are related to the animal’s performance during associative learning (*Gruart et al., 2006*). As illustrated in Figure S3, the slope of fEPSPs evoked by Schaffer collaterals stimulation at the CA3-CA1 synapse in 3-month-old wild-type mice was linearly related (*r* ≥ 0.74; *P* < 0.001) to the percentage of CRs across conditioning (slope = 0.64) and extinction (slope = 0.81) sessions, but not during habituation. Similar results were collected for 3-month-old Polμ^-/-^ animals (*r* ≥ 0.67; *P* < 0.001; slopes = 0.59 for conditioning and 0.41 for extinction sessions). Eighteen-month-old Polμ^-/-^ mice still presented a significant (*r* ≥ 0.74; *P* < 0.001) linear relationship between fEPSP slopes and the percentage of CRs, although with steeper slopes (0.85 for conditioning and 1.18 for extinction sessions) indicating a narrower range both for synaptic plasticity and for the increase in the number of conditioned responses (Figure S3D). The fact that the regression lines for acquisition and extinction sessions presented similar slopes suggests that the activity-dependent plasticity at the CA3-CA1 synapse functions as a continuum for both acquisition and extinction processes in young wild-type animals and young and old Polμ^-/-^ mice (*Gruart et al., 2006*). In contrast, 18-month-old wild-type mice presented no significant relationships between fEPSP slopes and the percentage of CRs for both conditioning (*r* = 0.49; *P* < 0.001) and extinction (r = 0.43; *P* = 0.181) sessions (Figure S3C).

**Paired-pulse facilitation in young and aged Polμ^-/-^ and wild-type mice**

Facilitation evoked by the presentation of a pair of pulses at a selected interval is assumed to be a model of short-term plasticity present in some excitatory synapses of the hippocampal circuit, including the CA3-CA1 synapse. Paired-pulse facilitation has been correlated with the presence of presynaptic circuits regulating neurotransmitter release (*Zucker and Regehr, 2002*). It is to be expected that at this type of synapse wild-type animals present a larger fEPSP response to the 2nd (with respect to the 1st) stimulus at short intervals. The optimal interval for the CA3-CA1 synapse in mice has been established as ≈ 40 ms (*Sahún et al., 2007*). As illustrated in Figure S5, the four experimental groups presented a significant (*F*_(15,135)_ = 11.394; *P* < 0.001) increase of the response to the 2nd pulse at short (20-100 ms) time intervals. No significant difference was observed between values collected from the four experimental groups (*F*_(15,135)_ = 0.261; *P* = 0.998). Thus, the experimental deletion of the Polμ did not significantly modify the paired-pulse facilitation characteristic of the CA3-CA1 synapse.

**Polμ^-/-^** **brain does not present significant alterations in DSB incidence, autophagy or improved telomere maintenance capacity**

First, wild-type and Polμ^-/-^ brains dissected from old mice (21-24 months) and prepared to analyze the incidence of DSBs, inferred from the 53BP-1 staining on sections (Figure S6A) and level of autophagy by measuring the expression level of some selected genes (Atg5l, Atg7l, Atg12l, Map1Lc3b, and Atg9a) critical for execution or regulation of autophagy (Figure S6B). Wild-type and Polμ^-/-^ brains did not show relevant differences in these studies. Furthermore, were analyzed for telomere length using real time quantitative PCR method (*Estrada et al., 2011*); relative telomere length ratio (T/S) evaluation of brain samples in comparison with reference human cells lines Tin2, LXSN and Tin 2-13 (*Kim et al., 1999*) demonstrated that there were no significant difference between wild-type and Polμ^-/-^ samples (Figure S6C; left panel). Q-FISH analysis (Figure S6D) confirmed that no improvement in telomere maintenance in Polμ^-/-^ brains could be demonstrated.

**Analyses of potential compensatory effects on the** **Polμ^-/-^** **brain phenotype**

qRT-PCR analyses were carried out both in wild-type and Polμ^-/-^ mice for the evaluation of the closest members of the DNA polymerase X family, Polβ and Polλ (Figure S6A), at different stages of aging (11, 14 and 19 months). Results are expressed as relative levels of both polymerases in relation with the wild-type values, at each stage (Figure S6E). Clearly no compensatory effect could be defined. In addition, brain extracts from wild-type and Polμ^-/-^ mice (21-24 months) were prepared and assayed for different activities.

**Evaluation of 8oxoG tolerance activity in brain extracts**

Figure S7A shows that evaluation with a conventional template-primer (Material and Methods; scheme in the upper part) rendered an equivalent gap-filling activity (position +16) in both samples, using ddCTP and several variations of divalent activator metal. This indicates that both extracts demonstrate a similar global catalytic activity. In parallel, evaluation of wild-type versus Polμ^-/-^ brain extracts from old (21-23 months) mice demonstrated a different capacity for resolving a premutagenic insertion as the presence of 8oxoG in the template (see scheme in the upper part) monitored as the appearance of the full-length (+34) product (see scheme of the reaction in Figure 4D). That full-length bypass product (in which 8oxoG was tolerated as template) was especially prominent (Figure S7B) when dATP (error-prone reaction) was provided in comparison with dCTP (error-free reaction); dGTP and dTTP were also incorporated (errors) and was mostly Polμ-dependent (Figure S7B, C). When rNTPs were used, the profile was quite comparable although the difference between wild-type versus Polμ^-/-^ brain extracts was more evident (Figure S7C). Globally, Polμ^-/-^ extracts induced a 3,5 fold combined (Σ dA/dG/dT) reduction in (+34 full-length product) with respect to wild-type extracts, when dNTPs were evaluated, and 5,7 fold combined (Σ rA/rG/rU) reduction when rNTPS we used. Just for comparison we carried out the direct gap-filling reaction with recombinant hPolμ enzyme (Figure S7D, E) using the same template, and comparing the efficiency using dNTPs (D) or rNTPs (E); the results were quite similar to those found with the wild-type brain extracts. Therefore we assume that brains from old wild-type animals have a significant mutagenic bypass activity in templates harboring 8xoG lesions, as the most representative oxidative damage, and that this mutagenic potential is importantly contribute by Polμ.

**Polμ^-/-^** **brain demonstrates an altered mitochondrial activity**

Although the molecular studies did not point in this direction, due to the remarkable role of mitochondrial function in oxidative stress and, therefore, potentially in aging we examined the activity status in old (23-28 month) Polμ^-/-^ or wild-type brain. Preparation of mitochondrial fractions and spectrophotometric activities of individual complexes were performed as described elsewhere (*Birch-Machin and Turnbull, 2001*). Evaluation of the different basic parameters of mitochondial activity was carried essentially as previously described (*Acín-Pérez et al., 2004*) and mitocondrial supercomplex study also as previously described (*Acín-Pérez et al., 2008*).

The analyses did not find significant differences neither in mitochondrial content (estimated by the citrate synthetase (CS) activity; Figure S8A) nor in the mtDNA copy-number (Figure S8B). However, the results clearly demonstrated that both in crude homogenates and mitochondria brain fractions, Polμ^-/-^ samples showed a better performance after evaluation of several relevant parameters (COX/CS, CII+CIII/CS and aconitase/CS ratios) in comparison with equivalent fractions obtained from wild-type brain (Figure S8C-E), expressed per mg protein. The effect is not global because evaluation of equivalent liver fractions did not reveal such a difference (Figure 5H). These results are in agreement with the described reduced ROS levels in Polμ^-/-^ mice (*Lucas et al., 2009; Escudero et al., submitted*) establishing a potential link between the delayed aging phenotype in Polμ^-/-^ brain and the classical hypothesis of aging centered in the structural cumulative damage produced by ROS, both in proteins and lipids as well is in DNA (*Hamilton, 2001*).

The structural organization of the mitochondrial respiratory complexes as four big independently moving entities connected by the mobile carriers CoQ and cytochrome c has been challenged recently (*Acín-Pérez et al., 2008*). Blue native gel electrophoresis reveals the presence of high-molecular-weight bands containing several respiratory complexes and suggesting an in vivo assembly status of these functional structures (respirasomes) proposing a novel structural organization model that accommodates these findings (*Acín-Pérez et al., 2008*). Mitochondrial OXPHOS complexes from wild-type and Polμ^-/-^ brain fractions, solubilized with digitonin (DIG), were separated in blue native gel electrophoresis (BNGE) and analyzed by western blot (Figure S8F) demonstrating no relevant differences in these supercomplexes assembly involving complex II (FpSDH), complex III (core2) and complex IV (COX1). Only mild moderate differences in the representation of complex III and IV in Polμ^-/-^ mitochondrial brain fractions were detected.

**Gene expression profile associated with the aging phenotype in Polμ^-/-^ brain**

Inflammation (*Krabbe et al., 2004*), electron transport (*Prolla et al., 2002; Lu et al., 2004*), and cell death (*Nakamura and Lipton, 2009*) are processes related to physiological aging, both in brain and in other organs (*Zhan et al., 2006*). With regard to the inflammation process, the brain of Polμ^-/-^ mice shows a highly decreased -although very variable inter-individual- expression of *Cox2*, a pro-inflammatory protein (*Williams and DuBois, 1996; Arslan and Zingg, 1996*). We also found that two components of the complement system ― *C5aR* and *C1qa* ― are down-regulated in the aged Polμ^-/-^ brain. However, there are no significant differences in the expression of C3aR and C5l2. We found 3 apoptosis-related genes, which are down-regulated in the aged Polμ^-/-^ brain. *Bid* (BCL-2 Interacting Domain, a modulator of pro-apoptotic function), is critically involved in the extrinsic cell death pathway (*Deng et al., 2003*), and its expression is highly decreased in the aged Polμ^-/-^ brain; *Egln3* (also known as *Phd3*, prolyl-hydroxylase domain protein 3) is a neuronal apoptosis-inductor (*Lipscomb et al., 1999; Straub et al., 2003; Lee et al., 2005*), and it also shows differential expression in the knockout brain. There is no detectable expression of *Egln3* in either wild-type or Polμ^-/-^ brain until the age of 16 months, when only the wild-type mice show detectable level of transcripts (we have represented the amount corresponding to 41 PCR cycles, since 40 is the last detection cycle); *Acin1* (Acinus; apoptotic chromatin condensator) is one of the targets of caspases during apoptosis implicated in nuclear changes (*Sahara et al., 1999*), and its expression is slightly (but not significantly) decreased in the knockout brain. As p53 is a transcription factor involved in apoptosis (*Levine, 1997*), we analyzed the expression of some of its targets (apart from Bid, directly recognized as a pro-apoptotic gene) ― namely *P21, P16, Perp,* and *Pten.* We did not find any relative down-regulation of these genes. Curiously, *P16* is under-expressed in young animals but overexpressed in old ones (Figure S9A); this clearly rules out that P16 could play a relevant role in the putative delayed brain aging phenotype in Polμ^-/-^ mice. Regarding the mitochondrial components (*Mrpl28, Mrps12, Timm17a, Ndufa10, Atp5a*), we did not find any modulated gene, indicating a delayed aging in Polμ^-/-^ brain.

Additionally, we also analyzed expression of selected genes with a demonstrated function in brain development or homeostasis (Figure S9B). Most of them (*Sox2, Snca, Hoxa3, Zic3, Hes1*) did not show variations in expression when adult Polμ^-/-^ and wild-type mice were compared, without or after paraquat (p) treatment (strong acute pro-oxidative treatment; Mateial and Methods). Only BMP4 showed an interesting response (Figure S9B). BMP4 has been clearly associated with age-related macular degeneration (AMD), mediating oxidative stress and senescence via Smad and p38 pathways *(Zhu et al., 2009*), impairment of endothelial function through cyclooxygenase-2 upregulation (*Tian et al., 2011*) and also in the inhibition of expansion of hippocampal subgranular zone (SGZ) precursors in comparison with subventricular zone (*Bonaguidi et al., 2008*). In a model of hypoxic-ischemic brain damage in newborn rats it has been demonstrated that bFGF has a neurorestoration effect, promoting expression of BMP4 in hippocampus and inhibiting apoptosis of neural cells (*Yin et al., 2009*). Finally, it has been proposed that BMP signaling mediates the effects of exercise on neurogenesis and cognition in the adult hippocampus. Elective exercise reduces levels of hippocampal BMP signaling before and during its promotion of neurogenesis and learning (*Gobeske et al., 2009*). Transgenic mice with decreased BMP signaling or wild-type mice infused with a BMP inhibitor both exhibit remarkable gains in hippocampal cognitive performance and neurogenesis, mirroring the effects of exercise. Conversely, transgenic mice with increased BMP signaling have diminished hippocampal neurogenesis and impaired cognition. The clear differences in BMP4 up-regulation upon acute oxidative stress challenge (Supplementary Results and Figure S9B) strongly suggest that Polμ^-/-^ brain could be less prompted to suffer premature senescence associated with BMP4.

**Differential gene expression studies reveals two gene networks altered in Polμ^-/-^** **brain**

Agilent microarray platform was used to compare brain expression profiles of old (18 m) wild-type and Polμ^-/-^ mice, without and with an acute treatment of paraquat (50mg/Kg; organs recovered after 7 h). Analysis of non-treated animals was previously described (Figure 4). Aiming to evaluate the involvement of a differential oxidative stress response in the described phenotype, Polμ^-/-^and wild-type mice were treated with paraquat and brain analyzed. It was demonstrated that 9.365 genes (38.7%) were deregulated (Figure S10A), but very few (0.03%) were differentially altered between Polμ^-/-^and wild-type mice (Figure S10A-C). This group of genes includes *Igfbp3, Lrrc46, Erdr1,* *Myo1g, Tmed4, NipSnap1* and *Ascc2. Igfbp3* has been previously demonstrated to be up-regulated in several cell systems as consequence of oxidative stress, mediating in amplification of hyperglycemic damage (*Yoo et al., 1999*); *Igfbp3* is down-regulated (0.8-fold) in Polμ^-/-^ mice resting brain. *Erdr1* (erythroid differentiation regulator 1) was modestly up-regulated (1.3-fold) in non-treated (basal) animals (Figure 3C). *Erdr1* has been defined as a proapototic or antimetastatic factor (*Kim et al., 2011*), but the most interesting correlation established derived for the seminal work of Sauvageau's group, who identified *Erdr1* as a HoxB4-like nuclear factor capable to promote self-renewal hematopoietic stem cells (HSCs) after forced overexpression (*Deneault et al., 2009*). Therefore, the modest overepresentation of *Erdr1* in Polμ^-/-^ mice could contribute to a better maintenance of tissue homeostasis. No obvious relationship could be established with the other modulated genes, and no other major player in oxidative stress control was revealed involved (Figures S9A and S10). Figure S10C shows the second gene network revealed, centered in around several Hox genes (*A3, B2, B3, B4* and *C4*), but with no established implication in brain aging**.**

Finally, functions included in both defined interaction networks, *Ptgg1* (securine) and *Erdr1*, show an expression modulation in Polμ^-/-^ mice that resembles the effect of overexpression of the neuroprotective gene (*WldS*), namely down-regulation of *Ptgg1* and up-regulation of *Erdr1* (*Gillingwater et al., 2006*). All together, results suggest a moderate increased damage resistance state in the Polμ^-/-^ mice.

**B. References included in the Supplementary Material**

- Acín-Pérez, R. *et al.*, *Mol. Cell* **13**, 805 (2004).
- Acín-Pérez, R. *et al.*, *Mol. Cell* **32**, 529 (2008).
- Ahnesorg, P., Smith, P., Jackson, S.P., *Cell*, **124**, 301 (2006).
- Aoufouchi, S. *et al.*, *Nucleic Acids Res*. **28,** 3684 (2000).
- Arslan, H., Zingg, H., *Prostaglandins* **52**, 463 (1996).
- Barnes, D.E., Stamp, G., Rosewell, I., Denzel, A., Lindahl, T., *Curr. Biol.* **8**, 1395 (1998).
- Bertocci, B., De Smet, A., Berek, C., Weill, J.C., Reynaud, C.A., *Immunity* **19**, 203 (2003).
- Bertocci, B., De Smet, A., Weill, J.C., Reynaud, C.A., *Immunity* **25**, 31 (2006).
- Birch-Machin, M.A., Turnbull, D.M., *Methods Cell Biol*. **65**, 97 (2001).
- Bonaguidi, M.A. *et al., J Neurosci*. **28**, 9194 (2008).
- Buck, D. *et al*., *Cell* **124**, 287 (2006).
- Capp, J.P. *et al.*, *Nucleic Acids Res.* **34**, 2998 (2006).
- Capp, J.P. *et al.*, *Nucleic Acids Res*. **35**, 3551 (2007).
- Chun, J.J., Schatz, D.G., Oettinger, M.A., Jaenisch, R., Baltimore, D., *Cell* **64,** 189 (1991).
- Cushman, J., Lo, J., Huang, Z., Wasserfall, C., Petitto, J.M. *Clin. Diagn. Lab. Immunol*. **10**, 13 (2003).
- Deneault, E. *et al.,* *Cell* **137**, 369 (2009).
- Deng, Y., Ren, X., Yang, L., Lin, Y., Wu, X., *Cell* **115**, 61 (2003).
- Domínguez-del-Toro, E. *et al*., *Eur. J. Neurosci.* **20**, 1945 (2004).
- Domínguez, O. *et al.*, *EMBO J.* **19**, 1731 (2000).
- Epel, E, *et al*., *Ann. N. Y. Acad. Sci.* **1032**, 208 (2004).
- Estrada, J.C. *et al*., *Cell Death Differ.* doi: 10.1038/cdd.2011.172 (2011).
- Evinger, C., Manning, K.A., Sibony, P.A., *Invest. Ophthalmol. Visual Sci.* **32**: 387 (1991).
- Frank, K.M. *et al*., *Nature* **396**, 173 (1998).
- Frank, K.M. *et al*., *Mol. Cell* **5**, 993 (2000).
- Gao Y. *et al*., *Cell* **95**, 891 (1998a).
- Gao, Y. *et al.*, *Immunity* **9**, 367 (1998b).
- Gericke, S. *Med. Hypotheses* **71,** 360 (2008).
- Gobeske, K.T. *et al.* *PLoS One* **4**, e7506 (2009).
- Gozalbo-López, B. *et al., Mol. Cell Biol*. **29**, 1266 (2009).
- Gruart, A., Muñoz, M.D., Delgado-Garcia, J.M., *J. Neurosci.* **26**, 1077 (2006).
- Gu, Y. *et al.*, *Immunity* **7**, 653 (1997).
- Gillingwater, T.H., *Hum. Mol. Gene*t. **15**, 625 (2006).
- Hamilton, K.K., *Clin. J. Oncol. Nurs.* **5**, 181 (2001).
- Holcomb, V.B., Vogel, H., Marple, T., Kornegay, R.W., Hasty, P. *Oncogene* **25**, 7159 (2006).
- Juárez, R., Ruiz, J.F., Nick McElhinny, S.A., Ramsden, D., Blanco, L., *Nucleic Acids Res*. **34**, 4572 (2006).
- Kan'o, M. *et al.*, *J. Radiat. Res. (Tokyo)* 48, 31 (2007).
- Karanjawala, Z.E., Murphy, N., Hinton, D.R., Hsieh, C.L., Lieber, M.R., *Curr. Biol*. **12,** 397 (2002).
- Karanjawala, Z.L., Hinton, D.R., Oh, E., Hsieh, C.L., Lieber, M.R., *DNA Repair (Amst)* **2**, 1429 (2003).
- Kim, S.H., Kaminker, P., Campisi J., *Nat Genet.* **23**, 405 (1999).
- Krabbe, K.S., Pedersen, M., Bruunsgaard, H., *Exp. Gerontol*. **39**, 687 (2004).
- Kugelberg, E., *Brain* **75**, 385 (1952).
- Kurimasa, A., *et al*., *Proc. Natl. Acad. Sci. U. S. A.* **96**, 1403 (1999).
- Lee, S. *et al*., *Cancer Cell*. **8**, 155 (2005).
- Lee, Y., McKinnon, P.J. *Neuroscience* **145,** 1365 (2007).
- Lee, K., Zhang, Y., Lee, S.E., *Nature* **454**, 543 (2008).
- Levine, J., *Cell* **88**, 323 (1997).
- Lieber, M.R., Ma, Y., Pannicke, U., Schwarz, K., *DNA Repair (Amst.)* **3**, 817 (2004).
- Lipscomb, E.A., Sarmiere, P.D., Crowder, R.J., Freeman, R.S., *J. Neurochem*. **73**, 429 (1999).
- Liu, Y. *et al.*, *Hum. Mutat*. **29**, 381 (2008).
- Lu, T. *et al*., *Nature* **429**, 883 (2004).
- Lucas, D. *et al*., *Eur. J. Immunol.* **35**, 1601 (2005).
- Lucas, D. *et al.*, *PLoS Genet.* **5**(2):e1000389 (2009).
- Ma, Y. *et al*., *Mol. Cell* **16**, 701 (2004).
- Mahajan, K.N., Nick McElhinny, S.A., Mitchell, B.S., Ramsden, D.A., *Mol. Cell. Biol.* **22**, 5194 (2002).
- Mao, Z., Bozzella, M., Seluanov, A., Gorbunova, V., *Cell Cycle* **7**, 2902 (2008).
- Moon, F. *et al.*, *Nat. Struct. Mol. Biol.* **14**, 45 (2007a).
- Moon, F. *et al.*, *DNA Repair (Amst)* **6**, 1709 (2007b).
- Moshous, D. *et al*., *Cell* **105**, 177 (2001).
- Nakamura, T., Lipton, S.A. *Apoptosis* **14**, 455 (2009).
- Natarajan, T., Berni, A., Marimuthu, K.M., Palitti, F., *Mutat. Res.* **642**, 80 (2008).
- Neema, M. *et al.*, *Hippocampus* **15,** 1057 (2005).
- Nick McElhinny, S.A. *et al.*, *Mol. Cell* **19**, 357 (2005).
- Nick McElhinny, S.A., Ramsden, D.A. *Mol. Cell Biol.* 23, 2309 (2003).
- Nussenzweig, A., *et al.*, *Nature* **382**, 551 (1996).
- Orii, K.E., Lee, Y., Kondo, N., McKinnon, P.J., *Proc. Natl. Acad. Sci. USA* **103**, 10017 (2006).
- Ouyang, H., *et al.*, *J. Exp. Med.* **186**, 921 (1997).
- Porras-García, E., Cendelin, J., Domínguez-de-Toro, E., Vozeh, F., Delgado-García, J.M., *Eur. J. Neurosci*. **21**, 979 (2005).
- Prolla, T.A., *Chem. Senses* **27**, 299 (2002).
- Rao, K.S., *Neuroscience* **145**,1330 (2007).
- Ren, K., Peña de Ortiz, S., *J. Neurochem*. **80,** 949 (2002).
- Rosidi, B. *et al*., *Nucleic Acids Res.* **36**, 1610 (2008).
- Ruiz, F. *et al.*, *Philos. Trans. R. Soc. Lond. B. Biol. Sci.* **356**, 99 (2001).
- Ruiz, F. *et al.*, *Nucleic Acids Res.* **32**, 5861 (2004).
- Sahara, S. *et al.*, *Nature* **401**, 168 (1999).
- Sahún, I. et al., *J. Neurosci.* **27**, 2253 (2007).
- Sharma, S., *Brain Res. Bull.* **73**, 48 (2007).
- Sonoda, E., Hochegger, H., Saberi, A., Taniguchi, Y., Takeda, S., *DNA Repair (Amst.)* **5**, 1021 (2006).
- Soulas-Sprauel, P. *et al.*, *Oncogene* **26**, 7780 (2007).
- Straub, A., Lipscomb, E.A., Yoshida, E.S., Freeman, R.S., *J. Neurochem*. **85**, 318 (2003).
- Sun, J.G., Han, S., Ji, H., Zheng, Y., Ling, S.C., *Zhejiang Da Xue Xue Bao Yi Xue Ban.* **36,** 161 (2007).
- Taccioli, G.E. *et al*., *Immunity* **9**, 355 (1998).
- Tian, X. *et al.* *Antioxid Redox Signal*, **16**, 363 (2011).
- Vyjayanti, V.N., Rao, K.S., *Neurosci. Lett*. **393,** 18 (2006).
- Wang, J., Ren, K., Pérez, J., Silva, S. Peña de Ortiz, A.J., *Learn. Mem*. **10,** 503 (2003).
- Williams, C.S., DuBois, R.N., *Am. J. Physiol.* **270**, G393 (1996).
- Yin, X.J. *et al*. *Zhonghua Er Ke Za Zhi*. **47**, 856 (2009).
- Zhu, M.A., Bogue, D.S., Lim, P., Hasty, D.B., Roth, *Cell* **86**, 379 (1996).
- Zhu, X., Deng, J., Xu, J., Hinton, D.R., *Aging* **1**, 740 (2009).
- Zucker, R.S., Regehr, W.G., *Annu. Rev. Physiol.* **64**, 355 (2002).
